# Intelligent Evaluation and Dynamic Prediction of Oyster Freshness with Electronic Nose Based on the Distribution of Volatile Compounds Using GC–MS Analysis

**DOI:** 10.3390/foods13193110

**Published:** 2024-09-28

**Authors:** Baichuan Wang, Xinyue Dou, Kang Liu, Guangfen Wei, Aixiang He, Yuhan Wang, Chenyang Wang, Weifu Kong, Xiaoshuan Zhang

**Affiliations:** 1 Beijing Laboratory of Food Quality and Safety, College of Engineering, China Agricultural University, Beijing 100083, China; b20193070600@cau.edu.cn (B.W.); 15856916264@163.com (K.L.); 2 Yantai Institute of China Agricultural University, Yantai 264670, China16678255906@163.com (Y.W.); 15318765715@163.com (C.W.); 3 School of Information and Electronic Engineering, Shandong Technology and Business University, Yantai 264005, China; guangfen.wei@sdtbu.edu.cn (G.W.);

**Keywords:** oysters, GC-MS, electronic nose, volatile components, freshness assessment, pattern recognition algorithms

## Abstract

The quality of oysters is reflected by volatile organic components. To rapidly assess the freshness level of oysters and elucidate the changes in flavor substances during storage, the volatile compounds of oysters stored at 4, 12, 20, and 28 °C over varying durations were analyzed using GC-MS and an electronic nose. Data from both GC-MS and electronic nose analyses revealed that alcohols, acids, and aldehydes are the primary contributors to the rancidity of oysters. Notably, the relative and absolute contents of Cis-2-(2-Pentenyl) furan and other heterocyclic compounds exhibited an upward trend. This observation suggests the potential for developing a simpler test for oyster freshness based on these compounds. Linear Discriminant Analysis (LDA) demonstrated superior performance compared to Principal Component Analysis (PCA) in differentiating oyster samples at various storage times. At 4 °C, the classification accuracy of the optimal support vector machine (SVM) and random forest (RF) models exceeded 90%. At 12 °C, 20 °C, and 28 °C, the classification accuracy of the best SVM and RF models surpassed 95%. Pearson correlation analysis of the concentrations of various volatile compounds and characteristic markers with the sensor response values indicated that the selected sensors were more aligned with the volatiles emitted by oysters. Consequently, the volatile compounds in oysters during storage can be predicted based on the response information from the sensors in the detection system. This study also demonstrates that the detection system serves as a viable alternative to GC-MS for evaluating oysters of varying freshness grades.

## 1. Introduction

As a significant source of human food, aquatic products are highly favored by consumers due to their abundant nutritional value and delightful taste [1]. Oysters, often referred to as the “milk of the sea”, are particularly rich in unsaturated fatty acids, proteins, vitamins, minerals, and notably, taurine [2,3,4,5]. However, oysters are prone to spoilage and deterioration during transportation and storage due to their high-water content, delicate muscle tissue, active endogenous enzymes, and microbial activity. These factors can lead to a decline in quality, a reduction in nutritional value, and even the production of harmful substances that raise food safety concerns [6,7,8]. Therefore, it is essential to assess the quality of oysters throughout the processing flow, with freshness being the most critical indicator of oyster quality [9].

Under the influence of microorganisms and enzymes, water products can generate volatile organic compounds (VOCs), the composition and concentration of which can serve as indicators of decay [10]. Gas chromatography–mass spectrometry (GC-MS) is commonly employed for detecting volatile gases in water products due to its advantages, including minimal sample requirements and high sensitivity, which enable the identification of specific compounds [11,12,13,14]. However, this conventional analytical method is time-consuming, complex, and requires skilled personnel for operation.

The electronic nose serves as an effective monitoring method, capable of comprehensively capturing and analyzing the overall characteristics and potential features of tested samples through the construction of a sensor array with specific identification capabilities and an advanced pattern recognition system. Meisam Vajdi et al. [15] utilized seven metal oxide gas sensors to develop an electronic nose for monitoring fish volatiles, complementing this with total viable count (TPC) and total volatile basic nitrogen (TVBN) detection methods. When modeling was performed using Principal Component Analysis (PCA) and a multilayer perceptron neural network (MLPNN), the accuracy of the test data reached 96.87%. Similarly, Parthasarathy Srinivasan et al. [16] conducted various auxiliary experiments and employed multiple recognition algorithms to construct the Shrimp-Nose model utilizing six metal oxide gas sensors under Fourier-transform infrared (FTIR) experimental conditions. Based on PCA, k-nearest neighbors (KNN), random forest (RF), and softmax regression algorithms for analysis, the softmax regression algorithm achieved decision accuracies of 96.29% for unfrozen samples and 95.73% for frozen samples.

Shoffi Izza Sabilla et al. [17] employed a greater number of sensors to develop a model with enhanced accuracy. They utilized an electronic nose equipped with eight MOS sensors to differentiate among three types of meat: chicken, pork, and beef. This was complemented by gas chromatography–mass spectrometry (GC-MS) and analytical methods such as Principal Component Analysis (PCA) and deep neural networks (DNNs). Their final model achieved an accuracy of 96.90%. Pei Li et al. [18] also increased the number of sensors, algorithms, and feature vectors to create a monitoring platform with ten gas sensors for assessing the freshness of fish meal. In conjunction with solid-phase microextraction gas chromatography–mass spectrometry (SPME-GC-MS) experiments, they employed various classification methods, including multilayer perceptron neural networks (MLPNNs), random forests (RFs), k-nearest neighbors (KNN), support vector classification (SVC), and Bayesian classifiers. Their findings indicated that the random forest method yielded the highest accuracy, achieving a classification accuracy of 90.7% when using integral values as feature vectors. Sun et al. [19] investigated the freshness of refrigerated tilapia by utilizing a gas sensor array in combination with a convolutional neural network (CNN) model, which achieved a prediction accuracy of 92.31% after optimization. Chen et al. [20] successfully predicted the freshness of salmon by integrating improved gas sensors with support vector machines.

The results of gas chromatography–mass spectrometry (GC-MS) provide detailed information regarding flavor, including the names and proportions of volatile compounds. though it is more time-consuming and requires skilled operation. In contrast, the electronic nose (E-nose) offers an overall assessment of aroma by utilizing a sensor array and pattern recognition algorithms. This allows for rapid and non-destructive aroma analysis, making it suitable for assessing food freshness. Consequently, the analysis of volatile compounds using GC-MS in conjunction with E-nose has been extensively documented [21,22,23,24]. While the E-nose and GC-MS have been used to assess the freshness of various aquatic products, their combined application to oysters remains unexplored. Given the unique composition and storage characteristics of oysters, applying these techniques presents specific challenges, such as accurately detecting subtle changes in volatile compounds during storage.

The present study systematically analyzed the changes in volatile compounds in oysters during various stages of storage. Qualitative and quantitative analyses of relevant volatile compounds throughout different storage periods were conducted using solid-phase microextraction (SPME) coupled with gas chromatography–mass spectrometry (GC-MS). Data on gas changes related to the deterioration of oyster freshness at temperatures of 4 °C, 12 °C, 20 °C, and 28 °C were collected using a self-designed electronic nose, leading to the development of a predictive model for oyster freshness. In the present study, the changes in volatile compounds in oysters during storage were systematically analyzed. The correlation between the content of amines, alcohols, sulfur compounds, and aldehydes representative markers associated with the freshness of aquatic products and the intensity of the electronic nose signals was examined. This research aims to provide insights into the integration of electronic nose technology with GC-MS for predicting oyster freshness during storage.

## 2. Materials and Methods

### 2.1. Sample Preparation

Before each experiment, live oysters with similar size were procured from a farm in Yantai and transported to the laboratory, where their shells were removed using specialized tools. The oyster meat, uniform in shape and size, was selected for the experiments, with each piece weighing approximately 20 g. Subsequently, 11 pieces of oyster meat, totaling about 220 g, underwent electronic nose testing at a specified temperature, completing six sets of parallel experiments. Concurrently, 2.5 g of oyster meat was placed in a headspace vial for gas chromatography testing, which was repeated three times to ensure the accuracy of the results.

### 2.2. E-Nose Analysis

#### 2.2.1. The Schematic of the Electronic Nose Architecture

**Figure 1:**
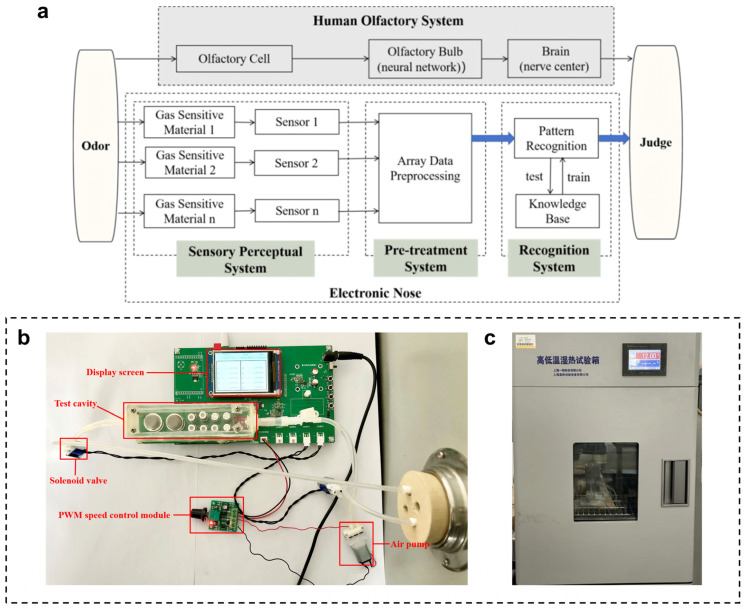
Electronic nose monitoring system. (**a**) General description of the human olfactory system and how it works with an electronic nose. (**b**) Electronic Nose development platform. (**c**) Thermostatic chamber.

As indicated in Figure 1a, the electronic nose is an apparatus analogous to the biological olfactory system, primarily consisting of three components: the gas sensor array, signal preprocessing, and mode identification. The gas sensor array functions similarly to the olfactory sensory cells in the biological olfactory system, while the signal preprocessing component corresponds to the olfactory receptor cells in the organism, and the mode identification serves a role akin to that of the biological cerebral processes.

During the procedure, upon receiving the gas information from the monitored sample, the gas sensor array converted this information into electronic signals, facilitating the efficient identification and classification of the samples by simulating the mechanisms of the human olfactory system. Following the reaction of various gases with the gas sensor materials, a specific feature-corresponding spectrum was generated, enabling qualitative analysis by these spectra to achieve accurate identification of the samples being measured.

#### 2.2.2. Construction of the Sensor Array

Various volatile components were emitted during the refrigeration of oysters, including significant amounts of aldehydes, amines, hydrocarbons, lipids, acids, ketones, and others. The volatility of these components changed systematically during the refrigeration process, reflecting the progression from freshness to spoilage. Considering the unique characteristics of the sensors and the gas emission patterns of oysters during refrigeration, this study selected ten types of metal oxide sensors with varying sensitivities to construct a sensor array. All sensors were sourced from the TGS series of FIGARO and the MQ series of Weisheng. This sensor array effectively leveraged the cross-sensitivity of the sensors to generate distinct fingerprints for each gas or odor. Consequently, this monitoring method was preferred over conventional single sensors, which often suffer from inadequate cross-sensitivity or selection issues. Table 1 provides detailed descriptions of the sensors along with their main characteristics.

#### 2.2.3. Experimental Design

A smart evaluation and dynamic predicting system was developed for a complete electronic nose monitor, designed based on a constructed sensor array. This system included an enclosed detection gas chamber, a gas sensor array, a humiture module, a main control chip, and an OLED display, as illustrated in Figure 1b. The system featured a red indicator that activates in the event of abnormal temperature and a green indicator for abnormal humidity. The OLED display was utilized to show various processes, such as initialization, cleaning, and data collection. The main control chip employed was the STM32F103C8T6 single-chip mini system, which utilizes a built-in A/D converter to transform analog signals into numerical signals representing gas density.

The detection gas chamber has been modified from a polypropylene storage box measuring 7.5 cm in height and 12.8 cm in diameter. This material was odorless, non-toxic, and exhibits excellent chemical and thermal resistance. Inlet and outlet ports have been created in the chamber, with a mini vacuum pump connected to each branch to facilitate the rapid intake and emission of gases. Additionally, two three-way solenoid valves have been installed at the back of the inlet mini pump to expedite the transition between the cleaning and data collection modes.

The monitoring of the electronic nose for oysters was conducted as follows: After preheating the electronic nose for one week, it was connected to the incubator (as illustrated in Figure 1c). Subsequently, oyster samples were placed into the incubators at the designated temperature before activating the air current control for continuous regular monitoring. Simultaneously, the upper computer was turned on to initiate data collection. Each monitoring period included a thorough cleaning lasting 10 min, followed by five data collection sessions. Both the data collection and short cleaning processes lasted 5 min, with a sampling frequency of 1 Hz. The access and cleaning loop for each individual sample were managed by the gas selection module on the testing equipment.

### 2.3. HS-SPME-GC-MS Analysis

#### 2.3.1. Instruments and Equipment

Agilent 8890/7010B (GC-MS/MS) instrument, with CTC multi-function injection system (including three-in-one injector, stirring oscillator, SPME ageing device, SPME Arrow injection tool, Agilent Technologies, Inc., Santa Clara, CA, USA; Extractor Smart SPME Arrows, 1.10 mm/120 µm DVB/carbon WR/PDMS, Agilent Technologies Technology Co., Ltd., Shanghai, China; 20 mL headspace thread sample bottle and threaded iron cap with silicone/PTFE pad, Shanghai ANPEL, HP-INNOWAX column, 30 m × OD 0.25 mm × film thickness 0.25 μm, Agilent Technologies Technology Co., Ltd., Shanghai, China.

#### 2.3.2. Detection of Volatile Substances

Solid phase microextraction involved accurately weighing each sample of 2.5 g in a 20 mL headspace bottle. The bottle was then filled with nitrogen for 10 s to remove oxygen, after which it was sealed immediately. The stirring oscillator module was heated to 80 °C with a stirring rate of 500 RPM, and the SPME Arrows DVB/carbon WR/PDMS extraction head was used for 20 min, followed by a gas phase inlet analysis for 3 min at 250 °C. The activation module temperature of the extraction head was set to 260 °C, the nitrogen purge was initiated, and the extraction head was activated for 3 min.

Gas chromatographic conditions included the use of an HP-INNOWAX column (30 m length, 0.25 mm outer diameter, and 0.25 μm film thickness). The inlet temperature was maintained at 250 °C, with samples injected in non-shunt mode. Helium served as the carrier gas at a flow rate of 1.0 mL/min. The programmed temperature rise began at an initial temperature of 50 °C, held for 2 min, then increased to 250 °C at a rate of 25 °C/min, followed by a further increase to 260 °C at 40 °C/min for 9 min.

Mass spectrometric conditions specify a chromatography–mass spectrometry interfaced temperature of 260 °C, an ion source temperature of 230 °C, and a quadrupole temperature of 150 °C. The ionization mode was electron impact (EI) positive, with an electron energy of 70 eV and a mass scanning range of 45 to 350 *m*/*z*.

#### 2.3.3. Identification of Volatile Compounds

In this study, the identification of volatile compounds was conducted using the NIST standard spectral library (Version 2.0g, build 4 December 2012). Specifically, compounds were identified by comparing their mass spectra and retention times with reference standards in the NIST library. Additionally, we utilized authentic standards (C-C) injected into the system to confirm the identification based on their mass spectra and retention times.

The relative content of each component was calculated using the area percentage method, which estimates the percentage content of each compound by calculating the ratio of the area of a single chromatographic peak to the total area of all peaks, as shown in Equation (1):

C_i_ = A_i_/∑A_i_ × 100% (1)

where A_i_ is the area of the chromatographic peak for component iii. In this calculation, we assume that all compounds have the same relative response intensity (i.e., *f*i = 1), without considering differences in response factors between compounds. This assumption simplifies the calculation and provides an approximate estimate of the relative content of each component.

### 2.4. Model Validation and Cross-Validation Approach

We employed 10-fold cross-validation to statistically validate the machine learning models used in this study. This method is widely recognized for its reliability in assessing model performance. The dataset was divided into ten equal parts, where nine parts were used for training the model and one part for testing. This process was repeated ten times, each time with a different part of the dataset used as the test set. The cross-validation results provided an average accuracy score, offering a more robust evaluation of the model’s predictive capabilities.

Additionally, the use of 10-fold cross-validation helped mitigate the risk of overfitting, as it ensured that each subset of the data was used for both training and validation. This approach provided a comprehensive assessment of model performance, highlighting its generalizability to new data. Accuracy was computed for each fold, and the final model performance was evaluated based on the mean values of this metric across all folds.

### 2.5. Statistics Analysis

After processing each independent sample, parameter analysis was conducted to further investigate the impact of processing and storage time on specific parameters. Two-factor analysis of variance (ANOVA) was performed using SPSS software (International Business Machines Corporation, version 20, New York, NY, USA) to examine these effects. Pearson correlation coefficient was utilized to explore potential associations between the parameters. Additionally, Duncan’s multivariate range test was employed to identify significant differences between means at the *p* ≤ 0.05 level.

## 3. Results and Discussion

### 3.1. Electronic Nose Detection

#### 3.1.1. Analysis of the Primitive Response Signal

**Figure 2:**
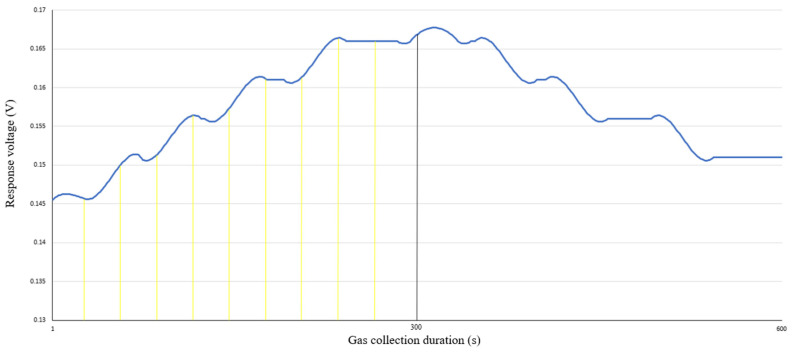
Schematic diagram of integral area in adsorption process.

The Trapezoidal Rule is employed to integrate the process of collecting gas samples from oysters. As illustrated in Figure 2, during the 600 s of the gas collection cycle, the first 300 s are dedicated to gathering gas sample data from the oysters, while the subsequent 300 s are allocated for cleaning the gas chamber with air. Consequently, only the integral area within the initial 300 s is computed in this context. The Trapezoidal Rule is a numerical integration technique utilized to estimate the value of a definite integral. The integration interval [*a*, *b*] is divided into several small trapezoids, which approximates the area under the curve and provides an estimated value of the definite integral.

**Figure 3:**
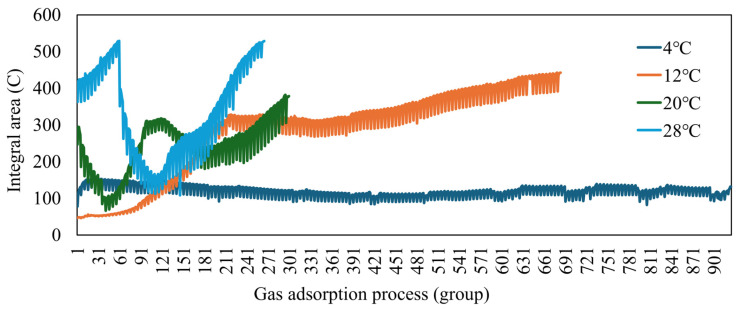
S1 sensor integral area line chart.

Specifically, the interval [*a*, *b*] is divided into *n* equal parts, each having a length of (*b* − *a*)/*n*. For instance, in Figure 3, the integration interval for this region spans the first 300 s, which are divided into 300 equal parts. The function values corresponding to the endpoints of each sub-interval are then obtained. The small intervals depicted in the figure are for illustrative purposes; in practice, the intervals are divided based on the irregularity of the area of the sub-divided intervals. The more irregular the division, the greater the number of intervals used. The heights at the two endpoints of each interval are multiplied by *h*, where *h* represents the response voltage at those endpoints. Subsequently, the area of the trapezoid in each sub-interval can be calculated. By summing the areas of the trapezoids across all sub-intervals, an approximate area under the entire interval [*a*, *b*] is obtained. This method is applied to the gas collection data from the oyster sample during the first 300 s, allowing for the calculation of the integral area of this adsorption process, as shown in the Equation (2).

(2) $$\int_{a}^{b}f\left(x\right)dx\approx \frac{h}{2\left[f\left(a\right)+2f\left(x_{1}\right)+2f\left(x_{2}\right)+\cdots +2f\left(x_{n-1}\right)+f\left(b\right)\right]}$$

Among them, *x*_1_, *x*_2_, …, *x_n_*_−1_ are the right endpoints of the *n* − 1 subintervals obtained by subdividing the interval [*a*, *b*].

In summary, the entire adsorption process was analyzed by calculating the integral area for oyster samples grouped in 5 min intervals. The integral areas of S1 at four different temperatures were aggregated and plotted as a line graph in Figure 3. It is evident that the change in integral area in response to the S1 sensor for oysters at 4 °C is not significant. However, as the temperature increases, the changes in integral area become more pronounced, although this does not facilitate direct analysis. A review of the literature suggests that the rate of volatile gas evaporation in oysters does not necessarily increase consistently with temperature variations. Consequently, the initial processing of the raw response signals does not provide a direct means for assessing oyster freshness.

#### 3.1.2. Signal Filtering Wave

During data collection, some of the gathered data may exhibit abnormalities due to environmental interference or other factors. Utilizing such abnormal data can have unpredictable effects on the entire system. To ensure system stability and mitigate the impact of environmental interference, it is essential to filter the raw data. Common filtering methods include arithmetic average filtering, Savitzky–Golay (SG) filtering, and recursive evaluation filtering. SG filtering is a linear filtering technique widely employed for smoothing data streams and reducing noise. This method is based on local polynomial least squares fitting in the time domain. The primary advantage of SG filtering is its ability to eliminate noise while preserving the shape and width of the signal. Compared to other filtering techniques, SG filtering offers distinct advantages, effectively minimizing noise while maintaining the trend and shape of the data. It allows for flexible adjustment of the polynomial fitting order, thereby accommodating various types of data with enhanced computational speed and reduced storage requirements. Consequently, SG filtering is utilized to process the raw oyster data, followed by feature extraction.

#### 3.1.3. Dimensionality Reduction of Data Based on PCA Algorithm

The choice of algorithms for different temperature conditions was guided by the nature of the data distribution at each temperature. Principal Component Analysis (PCA) and Linear Discriminant Analysis (LDA) were both employed to reduce dimensionality, but their performance varied depending on the dataset characteristics at different temperatures. At lower temperatures, the differences in the volatile compound profiles were subtler, requiring an approach like LDA, which maximizes the separation between classes. LDA was preferred in these cases because it is a supervised method that uses class labels to improve discrimination, making it more suitable for datasets where class separability is crucial. This enhanced the classification accuracy for samples stored at lower temperatures. Conversely, at higher temperatures, the variations in volatile compounds were more pronounced, leading to a dataset with clearer separation between classes. PCA, being an unsupervised method that focuses on capturing the variance in the data without relying on class labels, was sufficient for dimensionality reduction in these conditions. The use of PCA was favored here to preserve the overall data structure while reducing complexity, making it more efficient for datasets with naturally higher separability.

The experimental data, collected at four different temperatures, are presented in Table 2. Given that oysters spoil more rapidly at elevated temperatures, and with all other factors held constant, the number of gas data samples collected from oysters at higher temperatures is correspondingly diminished.

**Figure 4:**
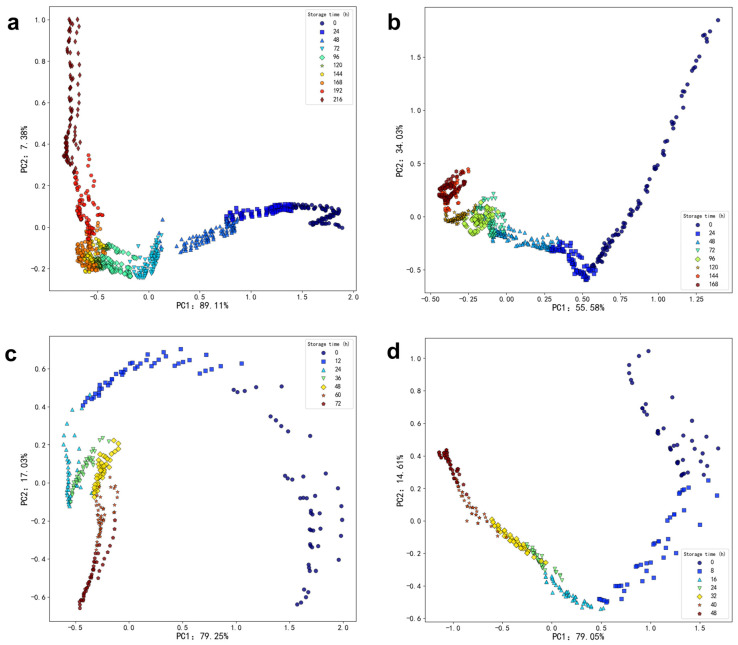
Visualization of PCA algorithm for feature extraction of oyster samples. (**a**) Gas samples from oysters in an environment of 4 °C. (**b**) Gas samples of oysters in an environment of 12 °C. (**c**) Gas samples of oysters in an environment of 20 °C. (**d**) Gas samples of oysters in an environment of 28 °C.

Through data collection, a substantial amount of experimental raw data undergoes initial preprocessing. Subsequently, the PCA algorithm is utilized to reduce the ten-dimensional data collected by sensors S1 to S10 to two dimensions. Figure 4 presents a scatter plot of the first and second principal components following the PCA transformation. The *x*-axis, PC1, corresponds to the first column of the reduced-dimensional data, while the *y*-axis, PC2, represents the second column. Each point in the plot corresponds to a sample feature, with points of the same color indicating features collected on the same storage time.

At 4 °C, the scatter plot generated by PCA indicates that the within-class distances increase each day, while the between-class distances decrease. This trend suggests that the classification criteria among the different categories are not significant, implying that the daily variation of volatile gases in oysters is relatively slow.

At 12 °C, the within-class distance on the first day of the experiment is small; however, there is a sudden increase in within-class distances on the first and second days, reflecting a greater amplitude of variation in the volatile gases of the oyster samples. In contrast, the within-class distances in the scatter plots for the final three days decrease, indicating that the various characteristics of the oysters tend to stabilize after spoilage. Compared to the scatter plot at 4 °C, the amplitude of volatile gas changes in oyster samples at 12 °C is greater.

At 20 °C, the within-class distance on the zero day is the largest, and as time progresses, the between-class distances of the scatter plots gradually decrease, reaching their smallest values in the last two days.

At 28 °C, the within-class distances in the scatter plots for the first two days are large and exhibit significant variation, suggesting that the amplitude of volatile gas changes in oyster samples is greater during this initial period.

It can be observed that as the temperature increases, the amplitude of gas variation in the oyster sample also increases. To achieve more accurate test results, an alternative method should be employed to analyze the oyster data.

#### 3.1.4. Dimensionality Reduction Based on LDA Algorithm

**Figure 5:**
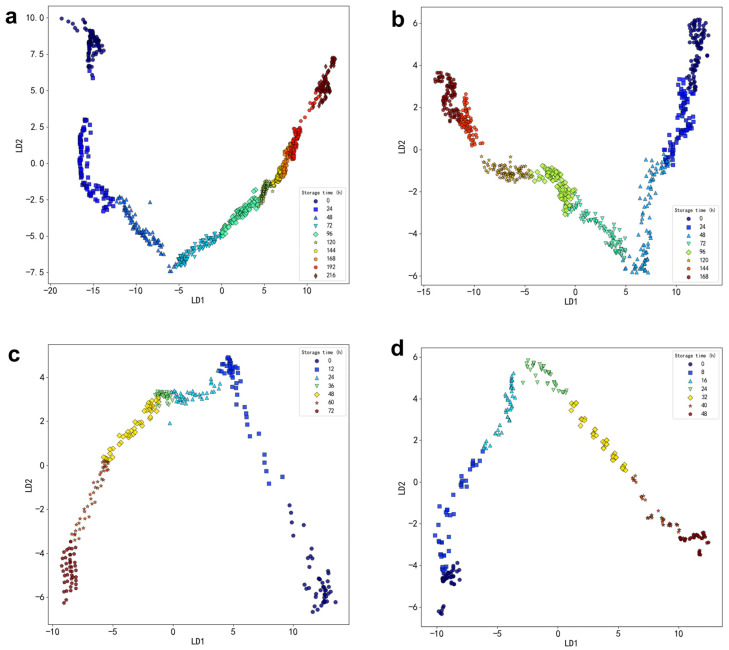
Visualization of LDA algorithm for feature extraction of oyster samples. (**a**) Gas samples from oysters in a 4 °C environment. (**b**) Gas samples from oysters in a 12 °C environment. (**c**) Gas samples from oysters in a 20 °C environment. (**d**) Gas samples of oysters in an environment of 28 °C.

Linear Discriminant Analysis (LDA) is a statistical method employed for dimensionality reduction, aimed at enhancing the similarity of samples within the same class while maximizing the dissimilarity between samples from different classes. LDA accomplishes this by maximizing the ratio of class-to-class distances to class-within-class distances, thereby identifying the optimal projection direction that preserves classification information in a lower-dimensional space. In Figure 5, the LDA-processed data of the oysters is presented, with different colors indicating samples collected on different days. Notably, the distribution of sample points in the figure exhibits significant changes over time, which may generally reflect the evolving characteristics of oysters during various storage periods.

Compared to PCA, LDA exhibits a relatively superior classification effect on the oyster data in a 4 °C environment. Within the storage time range of 72 to 192 h, both intra-class and interclass distances are very small, indicating that the gas production rate in oyster samples slowly changes within this time range.

In the LDA scatter plot for the 12 °C environment, the intra-class distances increase from the first to the third day when viewed from right to left, aligning with the conclusions drawn using the PCA algorithm in the previous section.

In the LDA scatter plot at 20 °C, both intra-class and interclass distances are relatively large at storage times of 0 and 12 h. At other storage times, both intra-class and interclass distances are relatively small.

In the LDA scatter plot at 28 °C, the interclass distance are relatively small during the storage time of 0 and 48 h, indicating that the gas emission rate of oyster samples changes slowly within this time range. At other storage times, the distance within the group was relatively large, indicating that the gas emission rate of oyster samples changes significantly within this time range.

The analysis of sensory sample records revealed that changes in the freshness of the oysters aligned with the conclusions derived from both Linear Discriminant Analysis (LDA) and Principal Component Analysis (PCA). By integrating these experimental findings, it was concluded that both LDA and PCA can serve as effective tools for evaluating the gas changes in oyster samples; however, the results obtained from LDA processing are found to be superior to those from PCA in this study. However, it is important to note that the choice between LDA and PCA depends on the specific dataset and application. While LDA may provide better classification performance in cases with well-separated classes and labeled data, PCA can be more appropriate when reducing dimensionality without specific class labels or when the primary goal is to preserve variance.

#### 3.1.5. A Classification Model Based on SVMs

Support vector machines (SVMs) are effective tools for addressing both classification and regression problems. The fundamental concept behind SVMs is to identify the optimal hyperplane that maximally separates different classes of data points. However, traditional linear SVMs struggle with non-linearly separable datasets. To overcome this limitation, the concept of kernel functions (KFs) was introduced, allowing for the mapping of original data into a higher-dimensional feature space, thereby facilitating linear separability in this extended space. The selection of an appropriate kernel function is a crucial step in the application of SVMs, as different kernel functions are better suited for various types of data distributions. Commonly used kernel functions include the linear kernel, polynomial kernel, and radial basis function (RBF) kernel. The linear kernel is appropriate for linearly separable or nearly linearly separable data, while alternative kernel functions are more effective for non-linear data.

In this study, the radial basis kernel function was employed, which is one of the most frequently used non-linear kernel functions, as illustrated in Equation (3).

(3) $$K\left(x,\;y\right)=exp\left(-\frac{{\left\|x-y\right\|}^{2}}{\left(2\cdot \sigma^{2}\right)}\right)$$

Specifically, *x* and *y* represent the input samples, and d(*x*, *y*) denote the Euclidean distance between *x* and *y*. Additionally, sigma refers to the standard deviation of the Gaussian distribution, while exp signifies the natural exponential function.

**Figure 6:**
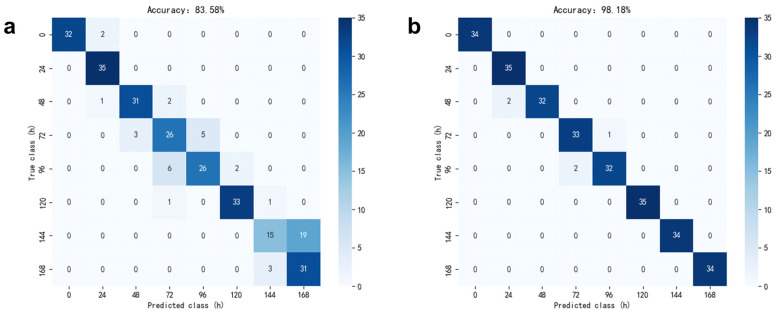
Confusion matrix. (**a**) Confusion matrix generated by SVM using PCA dimensionality reduction at 12 °C. (**b**) Confusion matrix generated by RF using LDA dimensionality reduction at 12 °C.

It is essential to evaluate the model’s performance on the test set, with accuracy being one of the most commonly used metrics, calculated by dividing the total number of correctly predicted samples by the total number of samples. The confusion matrix illustrates the correspondence between actual categories and the predicted categories generated by the model. Figure 6a presents a confusion matrix produced by the SVM algorithm following PCA dimensionality reduction on data processed at 12 °C. This matrix delineates the relationship between actual and predicted categories. Each row corresponds to actual values, while each column corresponds to predicted values. The matrix is 8 × 8, reflecting classifications based on storage time. In the confusion matrix, values along the diagonal indicate the number of correct predictions, whereas off-diagonal values represent incorrect predictions. Ideally, all predictions would lie on the diagonal, signifying that every prediction is accurate.

When using support vector machines (SVM) with the radial basis function (RBF) kernel, it is essential to standardize the data, as the RBF kernel is highly sensitive to the scale of the input features. Therefore, preprocessing the data through standardization is a necessary step before training the model. The RBF kernel can create complex decision boundaries, which may lead to high performance on the training set but poor generalization on the test set. Consequently, careful parameter selection and the use of cross-validation are imperative to mitigate the risk of overfitting. Additionally, the computational complexity associated with SVM should not be overlooked. While dimensionality reduction techniques can help reduce the data’s dimensionality, employing SVM with an RBF kernel can still result in significant computational demands, particularly when handling large-scale datasets. Thus, selecting appropriate optimization algorithms and computing resources is crucial for the successful application of SVM.

The SVM model, constructed using a dataset processed by the Principal Component Analysis (PCA) algorithm at a temperature of 12 °C, is illustrated in Figure 6a to predict the storage duration of oysters. This model demonstrates a classification accuracy exceeding 80%, indicating robust performance. However, there are several misclassified samples at storage durations of 72, 96 and 144 h, attributed to considerable overlap in sample points, as shown in Figure 4b.

Different dimensionality reduction methods were applied (Table 3) to the oyster sample data at varying temperatures, with an 60% training set and a 40% test set divided using SVM for the reduced data. The final processing results are summarized in the following table. Accuracy is employed to evaluate the results, revealing that the processing outcomes obtained through LDA for dimensionality reduction at the same temperature were significantly better than those achieved using PCA. Furthermore, the results obtained using the same processing method at different temperatures improved with increasing temperature. During the data dimensionality reduction stage, a comparison of scatter plots indicated that the scatter plot derived from LDA outperformed that of PCA, exhibiting smaller intra-class distances among the various scatter points. After LDA dimensionality reduction, the data features more effectively represented the changes in oyster freshness under different temperatures.

#### 3.1.6. Random Forest (RF)-Based Classification Model

Random forest (RF) is an ensemble learning method that generates final predictions by aggregating the outputs of multiple decision trees through a voting mechanism. This approach is particularly effective for handling high-dimensional data and capturing non-linear relationships. When applied to reduced datasets, random forest can effectively identify the primary features and make accurate classification or regression predictions.

One key difference between the random forest model and support vector machines (SVM) lies in the configuration of decision trees. Varying the number of decision trees trained can enhance the model’s accuracy, as demonstrated in Table 4, which summarizes the evaluation metrics for configurations with 10, 50, and 100 trees.

By comparing the prediction results of different numbers of decision trees in the table above, it is evident that the results for the oyster data in a 12 °C environment, after being reduced by Linear Discriminant Analysis (LDA) and processed through the random forest (RF) model, show significant improvements across various indicators as the number of decision trees increases. Conversely, the accuracy rates for other temperature conditions and algorithms after dimensionality reduction did not exhibit a significant improvement and, in some cases, even decreased. This phenomenon can be attributed to the fact that increasing the number of decision trees enhances the model’s complexity, allowing it to capture more non-linear relationships and patterns within the data [25]. If the underlying patterns in the dataset are sufficiently complex, augmenting the number of trees can aid the model in better fitting these patterns, thereby improving the accuracy rate.

Figure 6b illustrates the predicted storage time of oysters at 12 °C, utilizing a random forest model developed from data processed through the LDA algorithm. The model exhibits a classification accuracy greater than 95%, indicating its exceptional performance. A limited number of samples are inaccurately predicted for storage durations of 48, 72, and 96 h, which corresponds to the overlapping sample points for different storage times depicted in Figure 5b.

#### 3.1.7. Classification Model Evaluation and Optimization

By dividing the training set into 60% for training and 40% for testing, the accuracy of the classification predictions can be assessed. The specific data presented in Table 5 illustrate the distribution of training and testing samples, along with the samples that exhibited prediction errors. The classification predictions for each item indicate that the accuracy achieved after LDA dimensionality reduction is significantly higher than that obtained through PCA. Additionally, the scatter plot comparing the two methods in the previous section demonstrates that the classification performance of LDA is superior. When applying the same dimensionality reduction method to both RF and SVM, the prediction accuracies are nearly identical; however, the accuracy of the RF model improves consistently with an increase in the number of decision trees. Consequently, the optimized RF model outperforms SVM.

Optimizing the prediction model can significantly enhance its accuracy. In the random forest (RF) model, both the number of decision trees and the maximum depth of these trees can be adjusted to improve prediction accuracy. The depth of a decision tree directly influences the model’s complexity; deeper trees tend to have more intricate structures and must accommodate a greater number of features and rules, thereby increasing complexity. However, excessive complexity may compromise the model’s generalization ability, rendering it more vulnerable to noise and outliers. Consequently, it is crucial to strike a balance between accuracy and complexity when determining the depth of the decision tree to ensure that the model effectively fits the data while maintaining robust generalization capabilities. In previous experiments, various quantities of decision trees were tested, ultimately selecting 100 trees based on comparative analysis, as illustrated in Table 5, which presents a portion of the data following LDA dimensionality reduction at different depths.

The results indicated that altering the maximum depth of the decision tree did not impact the accuracy of the LDA dimensionality reduced data fed into the RF model. Conversely, the accuracy of the PCA dimensionality reduced data initially increased and then decreased with adjustments to the maximum depth of the decision tree. Therefore, when constructing an RF model, it is essential to select not only the appropriate number of decision trees but also to fine-tune the depth of the decision trees to ensure optimal overall model accuracy.

### 3.2. GC-MS Analysis Results

The gas volatilization from oysters stored at temperatures of 4 °C, 12 °C, 20 °C, and 28 °C was analyzed by GC-MS, yielding the volatile substances and their relative concentrations (Tables S1–S4). A summary of the substances detected under different storage conditions is as follows:

Storage at 4 °C: A total of 171 volatile compounds were identified across eight storage times. These included 25 alcohols, 19 aldehydes, 18 acids, 30 hydrocarbons, 15 ketones, one sulfur-containing compound, six nitrogen-containing compounds, 28 esters, three phenols, seven ethers, and 19 other compounds.

Storage at 12 °C: Across eight storage times, 173 volatile compounds were detected. This included 23 alcohols, 29 aldehydes, 12 acids, 45 hydrocarbons, 10 ketones, five nitrogen-containing compounds, 25 esters, three phenols, two ethers, and 19 other compounds.

Storage at 20 °C: A total of 137 volatile compounds were identified across seven storage times. These included 23 alcohols, 19 aldehydes, 13 acids, 33 hydrocarbons, nine ketones, five nitrogen-containing compounds, 16 esters, two phenols, two ethers, and 15 other compounds.

Storage at 28 °C: Across seven storage times, 161 volatile compounds were identified. These included 36 alcohols, 22 aldehydes, 17 acids, 25 hydrocarbons, 11 ketones, five nitrogen-containing compounds, 25 esters, and 19 other compounds.

The results indicate significant differences in both the types and concentrations of volatile substances in oysters, depending on the storage temperature and duration.

#### 3.2.1. Contents and Types of Volatile Substances in Oysters with Different Freshness

**Figure 7:**
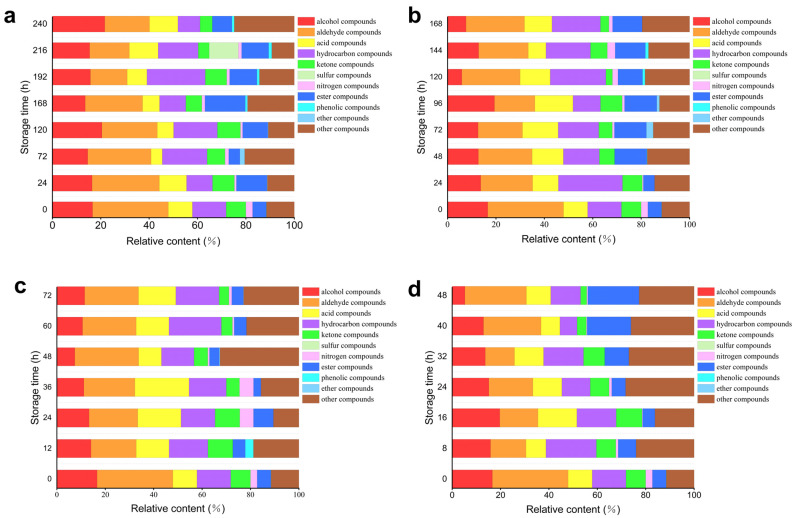
Relative contents of various volatile substances: (**a**) 4 °C, (**b**) 12 °C, (**c**) 20 °C, (**d**) 28 °C.

Figure 7 and Table 6 illustrate the relative contents of volatile compounds in oysters stored at various temperatures over time. As depicted in Figure 7a, at 4 °C, there is no significant difference in the relative content of alcohol, nitrogen-containing, and phenolic compounds as storage time increases. Additionally, the relative content of aldehyde compounds shows a decreasing trend, while the relative content of acid compounds exhibits an initial decrease followed by an increase. Additionally, the relative content of hydrocarbon, ketone, and ester compounds demonstrates an initial increase followed by a decrease. In Figure 7b, at 12 °C, it can be observed that the relative content of alcohol compounds tends to decrease with increasing storage time. Aldehyde and hydrocarbon compounds display a trend of initially decreasing followed by an increase in relative content during storage. The relative content of acid compounds shows an initial increase followed by a decrease. There are no significant differences in the relative content of ketone, nitrogen-containing, and ester compounds. In Figure 7c, at 20 °C, it is evident that the relative content of alcohol substances decreases initially and then increases during the storage process. The relative content of ketone substances increases initially and then decreases over time. The trends for acid substances and nitrogen-containing compounds also show an initial increase followed by a decrease during storage. No significant differences are observed in the relative content of aldehyde, hydrocarbon, and ester substances. In Figure 7d, at 28 °C, the relative content of aldehyde substances initially decreases before increasing again during the storage process. In contrast, hydrocarbon substances exhibit an initial increase followed by a decline. The relative content of ester substances shows a significant increase, whereas alcohol substances, ketone substances, and nitrogen-containing compounds demonstrate a decreasing trend throughout storage. Notably, no significant difference was observed in the relative content of acid substances, indicating that these compounds may not be key markers for freshness assessment in this context. Despite the numerous volatile organic compounds identified and tracked over time, the limited variation in acid compounds suggests that other pathways, such as the formation of heterocyclic compounds like cis-2-(2-Pentenyl) furan, play a more crucial role in the degradation process.

**Figure 8:**
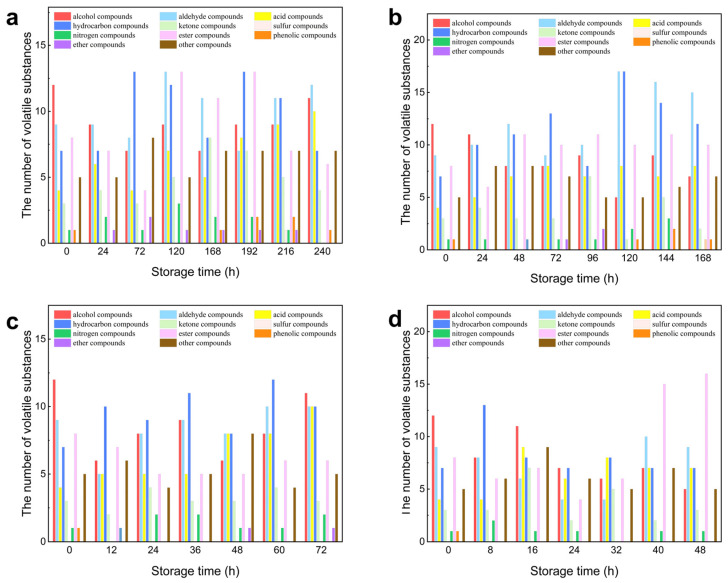
Quantity of various volatile substances in oysters with different storage time levels: (**a**) 4 °C; (**b**) 12 °C; (**c**) 20 °C; (**d**) 28 °C.

Figure 8 and Table 7 illustrate the comparison of volatile compounds in oysters at various storage temperatures. In Figure 8a, at 4 °C, the diagram reveals that during storage, oysters contain a substantial number of alcohols, hydrocarbons, and esters, which indicates their significant role in the volatile substances present in oysters. Conversely, the levels of phenols and ethers are relatively low. In Figure 8b, at 12 °C, it can be observed that the storage process results in a higher concentration of alcohols, hydrocarbons, and esters, further underscoring their importance in the volatile profile of oysters. Similarly, phenolic and ether compounds remain at lower concentrations. Figure 8c, at 20 °C, shows an increased presence of hydrocarbon compounds, along with relatively high levels of alcohols, aldehydes, and acids. In contrast, nitrogen-containing compounds, phenolic compounds, and ether compounds are found in lower quantities. Lastly, Figure 8d, at 28 °C, indicates that the storage process leads to a notable increase in ester compounds, alongside significant amounts of hydrocarbons, alcohols, aldehydes, and acids. Conversely, nitrogen-containing and ketone compounds are present in lower quantities.

**Figure 9:**
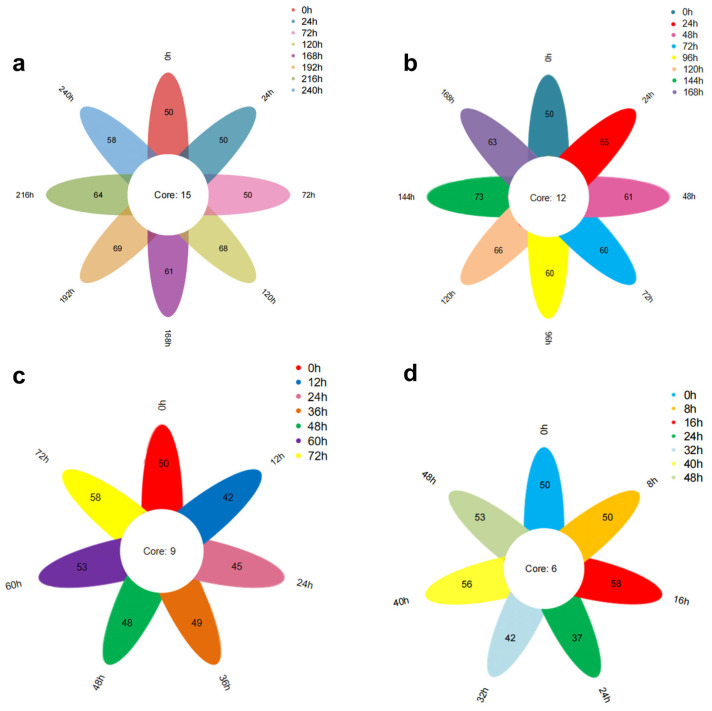
Petalvane diagram of volatile substances: (**a**) 4 °C, (**b**) 12 °C, (**c**) 20 °C, (**d**) 28 °C.

Figure 9a illustrates the Venn diagram depicting the total number of volatile substances in oysters stored at 4 °C over various periods. The data reveal that eight oyster samples with differing levels of freshness were identified to contain 50, 50, 50, 68, 61, 69, 64, and 58 types of volatile substances. Notably, these samples share a common set of fifteen volatile substances, which include cis, cis-7,10-Hexadecadienal, 2,4-Heptadienal (E,E), Benzaldehyde, 2,6-Nonadienal (E,Z), 2,4-Decadienal (E,E), 3-(Pent-1-en-1-yl)benzaldehyde, Tetradecanoic acid, n-Hexadecanoic acid, and 2-Methyl-1-nonene-3-yne. Figure 9b presents the Venn diagram for the total number of volatile substances in oysters stored at 12 °C over different periods. The figure indicates that eight samples with varying freshness contained 50, 55, 61, 60, 60, 66, 73, and 63 types of volatile substances. These samples share a common set of twelve volatile substances, including 2,6-Cyclooctadien-1-ol, Eicosen-1-ol, and cis-9.

Figure 9c illustrates the Venn diagram depicting the total number of volatile substances in oysters stored at 20 °C over various periods. The figure reveals that seven distinct freshness samples of oysters were identified, containing 50, 42, 45, 49, 48, 53, and 58 types of volatile substances. Notably, these samples shared a common set of nine volatile substances: Ethanol, 2-(dodecyloxy); Eicosen-1-ol, cis-9-; 2,6-Cyclooctadien-1-ol; 2,4-Heptadienal, (E,E)-; Tetradecanoic acid; n-Hexadecanoic acid; 1,5-Cyclooctadiene, 3-(1-methyl-2-propenyl)-; and 3,5-Octadien-2-one. Figure 9d presents the Venn diagram for oysters stored at 28 °C over various periods. From this figure, it can be observed that seven different freshness samples of oysters were identified, with 50, 50, 58, 37, 42, 56, and 53 types of volatile substances. These samples shared a common set of six volatile substances: Eicosen-1-ol, cis-9-; Tetradecanoic acid; n-Hexadecanoic acid; Dodecanoic acid; and cis-2-(2-Pentenyl) furan. The microbial influence during storage leads to fat oxidation and protein hydrolysis, resulting in the loss of older substances and the generation of new ones. Consequently, the number of common volatile substances in oysters with varying freshness levels is relatively small at each temperature.

Figure 9 illustrates the impact of storage time on the total volatile compounds under different temperature conditions (28 °C, 20 °C, 12 °C, 4 °C). The results indicate that the quantities of total volatile compounds at each temperature are 6, 9, 12, and 15, respectively, following a clear arithmetic sequence. The data reveal a decreasing trend in total volatile compounds as storage temperature increases.

This indicates that as the temperature increases, the biochemical reactions of volatile compounds become more extensive and complex.

#### 3.2.2. Analysis of Volatile Substances in Oysters with Different Freshness

Alcohols are the most representative volatile organic compounds produced during the storage of oysters. The types and relative percentages of alcohols in oysters vary at different stages of spoilage. According to GC-MS analysis of oysters stored at various temperatures (4, 12, 20, and 28 °C), 11, 20, 20, and 34 different alcohols were detected, respectively. During storage, the content of alcohols remained relatively high. Among these alcohols, Styrene, 1-Hexadecanol, Ethanol, 2-(tetradecyloxy)-, 1-Octadecanol, and methyl ether were only present in the early stages of storage. In contrast, 1-Ethynylcyclododecanol, Cyclohexanol, 5-methyl-2-(1-methylethenyl)-, 6-Pentyltetrahydro-2H-pyran-2-ol, and others were formed in the later stages of storage. Myrtenol, which has a grass, wood, and camphor-like aroma, exhibited particularly noticeable changes at 4 °C, with both its absolute and relative contents increasing significantly. At 12 °C, the absolute content of Myrtenol also increased markedly; however, due to the rise in other more volatile substances, the relative content of Myrtenol stabilized. Alcohols are generally formed through the reduction of aldehydes and ketones, as well as the oxidation of lipids, with a typically high threshold. Among these, unsaturated alcohols, such as 1-octene-3-ol, contribute to the fishy taste commonly associated with aquatic products. An increasing body of research indicates that unsaturated alcohols are produced through the oxidation of unsaturated fatty acids, imparting a mushroom-like flavor to fresh seafood [26]. The odor threshold of unsaturated alcohols is generally lower than that of saturated alcohols, significantly influencing the odor properties of oyster meat.

Aldehydes and ketones exhibit relatively high concentrations and low thresholds during storage, which significantly contributes to flavor [27]. Aldehydes are primarily formed [28] from unsaturated fatty acid hydroperoxides through the action of lipoxygenase, a crucial enzyme in food production that generates a variety of oxidative flavors. These aldehydes possess strong aromas reminiscent of grass, fat, and fruit [29]. Ketones, on the other hand, are unstable intermediate compounds typically produced by the degradation of amino acids and the thermal oxidation of unsaturated fatty acids. They can subsequently be oxidized or reduced into corresponding alcohols, which impart unique fruit flavors [30]. At storage temperatures of 4, 12, 20, and 28 °C, a total of 19, 29, 16, and 23 types of aldehydes, as well as 15, 10, 8, and 11 types of ketones, were detected in oysters at different storage durations. Notably, compounds such as benzaldehyde, 4-ethyl-, 2, 4-decadienal (E,Z)-, bicyclo [6.1.0]non-4-ene-9-carbaldehyde, 1-cyclohexene-1-acetaldehyde, and α,2-dimethyl- were identified exclusively in the later stages of storage, along with ketones like 2-tridecanone and 2-nonanone.

Fatty acids are often characterized by their aromatic and sweet notes; however, their impact on overall flavor is minimal due to their higher thresholds [31]. The primary substances identified during storage include acetic acid, caprylic acid, dodecanoic acid, tetradecanoic acid, pentadecanoic acid, and palmitic acid, among others. Acetic acid is the principal contributor to rancidity, which becomes particularly pronounced when its concentration exceeds 1%. Octanoic acid imparts a greasy and creamy taste, detectable at all storage temperatures, and is typically produced during the later stages of storage.

Composed of saturated fatty alcohols and lower levels of saturated fatty acids, most esters emit fruity and floral notes that effectively mask the bitter taste of amino acids and the pungent odor of fatty acids. A total of 28 esters were detected at 4 °C, with the relative content of esters being highest after 168 h of storage. At 12 °C and 20 °C, 21 and 13 types of esters were detected, respectively, while at 28 °C, a total of 27 esters were identified. At 12 °C, the absolute content of esters exhibited an increasing trend, although the relative content began to decline after the fifth day due to the production of additional substances. Conversely, at 20 °C and 28 °C, the absolute content of esters remained low, and the trends in relative content were not clearly defined, likely due to interference from microorganisms and changes in pH.

At all temperatures, the types and relative contents of phenolic and ether compounds are minimal. At 28 °C, no phenols or ethers were detected.

Various kinds of hydrocarbons are present during the storage process, but their relative content remains low. At temperatures of 4, 12, 20, and 28 °C, 30, 46, 31, and 34 types of hydrocarbons were detected, respectively. At 4 °C and 20 °C, the content of hydrocarbon compounds initially increased and then decreased, reaching peak levels at 192 h and 60 h, respectively. At 12 °C, the content of hydrocarbons showed a trend of first decreasing and then increasing. Conversely, at 28 °C, the content of hydrocarbons fluctuated significantly and the trend is irregular. Among these hydrocarbons, compounds such as Styrene, 1,3-Cyclooctadiene, and Allylidenecyclohexane were only detected in the early stages of storage. In contrast, substances like Pentadecane, 1H-Indene, 1-ethylideneoctahydro-, trans-Cyclohexene, and 3-ethyl appeared in the later stages of storage. Due to their higher odor threshold, hydrocarbons exert less influence on food odor [32]. However, under certain conditions, hydrocarbons can be converted into aldehydes and ketones, which may be responsible for the fishy odor observed in aquatic products [33].

Under all temperature conditions, the relative content of nitrogen-containing compounds remains low, while sulfur-containing compounds are only detected at 4 °C. At temperatures of 4, 12, 20, and 28 °C, a total of 6, 4, 5, and 7 types of nitrogen-containing compounds were identified, respectively. The primary nitrogen-containing compounds include oxime-, methoxy-phenyl-, and 2-(E)-hexen-1-OL, as well as (4S)-4-amino-5-methyl-. The decomposition of proteins predominantly generates nitrogen-containing compounds through the action of microorganisms and enzymes. Notably, only one sulfur-containing compound was detected in the volatile substances of oysters at 4 °C. These compounds were not present in particularly fresh oyster samples but became apparent with extended storage. Most sulfur compounds, which are characterized by aromas reminiscent of onion, cabbage, boiled sulfur, or rotten eggs, have low thresholds that significantly influence the overall flavor of the food.

The other compounds identified primarily include furan compounds, pyridine compounds, and various additional compounds. GC-MS analysis of oysters stored at different temperatures—4, 12, 20, and 28 °C—revealed the detection of 19, 17, 12, and 21 types of nitrogen compounds, respectively. Among the furan compounds, 2-ethyl-, cis-2-(2-pentenyl) furan was found to be the most abundant, characterized by a strong fleshy, burnt, and sweet aroma, as well as a very low aroma threshold, which contributes to an appealing fragrance. The Maillard reaction, which occurs between amino acids and reducing sugars, is one of the key processes responsible for the development of meat flavor. This complex reaction yields numerous important flavor compounds, including furan, pyrazine, pyrrole, and other heterocyclic compounds [34]. Furan, 2-pentyl- is primarily generated through the oxidation and degradation of lipids during the heating process. It is commonly found in cooked meat and meat products, imparting a baked flavor along with notes of beans, fruit, green, and similar vegetable flavors [35]. In low concentrations, furan, 2-ethyl- significantly influences the odor of oysters, contributing both burnt and sweet aromas [36].

### 3.3. Correlation of Various Volatiles with Sensor Arrays

**Figure 10:**
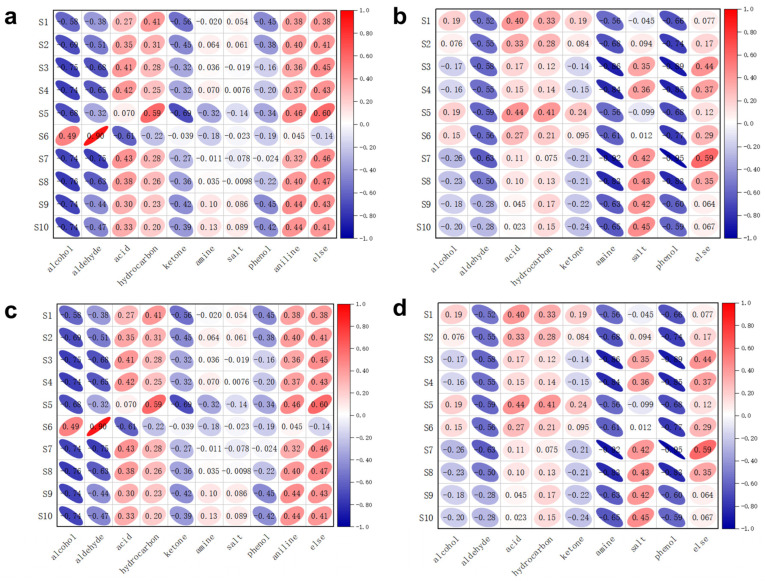
Correlation between volatile compound classes and sensor array: (**a**) 4 °C; (**b**) 12 °C; (**c**) 20 °C; (**d**) 28 °C.

From Figure 10, it is evident that at a temperature of 4 °C, aldehydes and nitrogen-containing compounds exhibit a strong linear relationship with the sensor response, whereas other volatile substances in the ‘others’ category demonstrate relatively weak linear correlations with the sensor response. Aldehyde compounds display high correlation coefficients with sensors 1, 2, 3, 4, 5, 9, and 10. In contrast, nitrogen-containing compounds show significant correlations with sensors 3, 7, and 8. Furthermore, from Table 3, it can be observed that phenolic substances at a temperature of 12 °C exhibit substantial and robust correlations with sensors 1 and 2.

After a comprehensive analysis of the correlation between volatile substances and the sensor array at various temperatures, the results indicated that sensors 1, 2, and 6 exhibited a higher association with amine substances. As shown in Table 1, these three sensors effectively detect nitrogen−containing compounds, demonstrating a high degree of consistency. Furthermore, sensors 1 and 2 displayed a significant correlation with sulfur-containing substances, suggesting that their target gases include sulfides such as hydrogen sulfide. Alcohol substances were found to have a strong correlation with sensors 7, 8, and 9, which consistently detected organic solvents like alcohol. Phenolic substances primarily correlated with sensors 1, 2, and 6, aligning with their high association with nitrogen-containing compounds; this indicates that this type of gas is not confined to a single category but exists in various forms. Aldehyde and ketone substances demonstrated significant correlations with sensor 9, which targets components such as organic solvents, showcasing good adaptability in this context. Hydrocarbon substances primarily involved sensors 1, 5, and 7; these sensors detected target gases including propane, methane, and iso-butane, illustrating strong compatibility in this regard. Additionally, a clear correlation was observed between ester compounds and sensors 6 and 8, while ether compounds were more closely related to channels 3, 5, and 6. Other types of compounds relied more on detection from channels 2, 9, and 10.

### 3.4. Discussion

Regarding the significance of replacing GC-MS with an electronic nose (E-nose), we have expanded the discussion to compare the advantages and limitations of both techniques. The E-nose, as a non-destructive, real-time, and cost-effective tool, offers significant benefits in monitoring the storage quality of fresh products like oysters. In contrast, while GC-MS provides high precision and specificity in detecting volatile organic compounds (VOCs), its high costs, operational complexity, and the need for skilled personnel limit its feasibility for on-site testing and large-scale applications.

Recent studies, such as those by Shi et al. [37] and Yi and Xie [38], have demonstrated the high sensitivity of the E-nose in monitoring changes in VOCs during storage. When combined with machine learning algorithms, the E-nose can effectively predict spoilage and quality degradation in seafood. This further highlights the potential of the E-nose in oyster storage quality monitoring.

Furthermore, integrating the E-nose with machine learning techniques enhances its predictive capabilities. The E-nose hardware collects VOC signals through gas sensors, and pattern recognition algorithms, like support vector machines (SVMs) and neural networks (NNs), are employed for analysis. These technologies significantly improve detection accuracy and efficiency. Recent studies [39,40] have shown the successful application of the E-nose combined with machine learning in quality detection of various food products, including fruits, vegetables, and meats. In the context of oyster storage monitoring, the E-nose, together with advanced machine learning techniques, could rapidly, economically, and effectively predict and detect quality changes, particularly in identifying specific VOC signal patterns.

## 4. Conclusions

This study demonstrated the effectiveness of using an electronic nose (E-nose) with gas chromatography–mass spectrometry (GC-MS) for assessing oyster freshness. By integrating the E-nose with machine learning algorithms such as support vector machines (SVMs) and random forest (RF), we achieved rapid and reliable freshness predictions. Linear Discriminant Analysis (LDA) outperformed Principal Component Analysis (PCA) in classification accuracy, particularly at higher storage temperatures, indicating its suitability for distinguishing subtle variations in volatile compounds during spoilage. Significant changes in volatile compounds, including an upward trend in cis-2-(2-Pentenyl) furan, suggest potential markers for developing simpler freshness tests. This integration provides a cost-effective and non-destructive alternative to traditional GC-MS, enabling real-time quality monitoring suitable for large-scale and on-site applications. The integration of E-nose and machine learning provides a promising alternative to traditional GC-MS analysis, offering a cost-effective and non-destructive approach for real-time monitoring of seafood quality. Future research could further refine the E-nose’s sensitivity to specific volatile markers and explore its application across other seafood products, contributing to improved food quality control and safety.

## Figures and Tables

**Table 1:** The sensor specifications utilized in the Oyster-Nose model.

S. No	Sensor Types	Volatile Compounds	Detection Range (ppm)
S1.	TGS2602	Ammonia, hydrogen sulfide, and toluene	1~30
S2.	TGS2603	Amine series, hydrogen sulfide, etc.	1~10
S3.	TGS2612	Methane, propane, isobutane, etc.	500~10,000
S4.	TGS2630	Refrigerant gas	1000~10,000
S5.	MQ137	Ammonia and amine compounds	5~500
S6.	MQ135	Ammonia gas, sulfide, benzene series vapor	10~1000
S7.	TGS2611	Ethanol, hydrogen, isobutane, methane	500~10,000
S8.	TGS2610	Ethanol, hydrogen, methane, isobutane/propane	500~10,000
S9.	TGS2620	Organic solvents, alcohol, etc.	50~5000
S10.	TGS2600	Carbon monoxide, hydrogen	1~30

**Table 2:** Oyster sample collection quantity.

Temperature Setting	Collection Duration	Effective Number of Detection Cycles	Quantity of Data Set
4 °C	216 h	185	925 EA
12 °C	168 h	137	685 EA
20 °C	72 h	60	300 EA
28 °C	48 h	53	265 EA

**Table 3:** SVM evaluation report.

Oyster Storage Temperatures	Dimensionality Reduction Method	Classification Labels (by Detection Hours)	Number of Training Samples (Groups)	Number of Test Samples (Groups)	Number of Predicted Incorrect Samples (Groups)	Accuracy
4 °C	PCA	0–216 h	555	370	53	80%
4 °C	LDA	0–216 h	555	370	22	94%
12 °C	PCA	0–168 h	411	274	49	82%
12 °C	LDA	0–168 h	411	274	11	96%
20 °C	PCA	0–72 h	180	120	8	93%
20 °C	LDA	0–72 h	180	120	3	98%
28 °C	PCA	0–48 h	159	106	14	87%
28 °C	LDA	0–48 h	159	106	2	98%

**Table 4:** RF evaluation report form.

Oyster Storage Temperatures	Dimensionality Reduction Method	Number of Decision Trees	Classification Labels (by Detection Hours)	Number of Training Samples (Group)	Number of Test Samples (Group)	Number of Predicted Incorrect Samples (Group)	Accuracy
4 °C	PCA	10	0–216 h	555	370	44	88%
4 °C	LDA	10	0–216 h	555	370	22	94%
12 °C	PCA	10	0–168 h	411	274	38	86%
12 °C	LDA	10	0–168 h	411	274	5	98%
20 °C	PCA	10	0–72 h	180	120	10	92%
20 °C	LDA	10	0–72 h	180	120	7	94%
28 °C	PCA	10	0–48 h	159	106	12	89%
28 °C	LDA	10	0–48 h	159	106	0	100%
4 °C	PCA	50	0–216 h	555	370	48	87%
4 °C	LDA	50	0–216 h	555	370	18	95%
12 °C	PCA	50	0–168 h	411	274	41	85%
12 °C	LDA	50	0–168 h	411	274	5	98%
20 °C	PCA	50	0–72 h	180	120	8	93%
20 °C	LDA	50	0–72 h	180	120	7	94%
28 °C	PCA	50	0–48 h	159	106	8	92%
28 °C	LDA	50	0–48 h	159	106	0	100%
4 °C	PCA	100	0–216 h	555	370	48	87%
4 °C	LDA	100	0–216 h	555	370	18	95%
12 °C	PCA	100	0–168 h	411	274	41	85%
12 °C	LDA	100	0–168 h	411	274	5	98%
20 °C	PCA	100	0–72 h	180	120	8	93%
20 °C	LDA	100	0–72 h	180	120	6	95%
28 °C	PCA	100	0–48 h	159	106	8	92%
28 °C	LDA	100	0–48 h	159	106	0	100%

**Table 5:** RF Optimization evaluation report form.

Oyster Storage Temperatures	Dimensionality Reduction Method	The Maximum Depth of Each Decision Tree	Classification Labels (by Detection Hours)	Number of Training Samples (Groups)	Number of Test Samples (Groups)	Number of Predicted Incorrect Samples (Groups)	Accuracy
12 °C	LDA	5	0–168 h	411	274	5	98%
12 °C	LDA	7	0–168 h	411	274	5	98%
12 °C	LDA	10	0–168 h	411	274	5	98%
12 °C	PCA	5	0–168 h	411	274	49	82%
12 °C	PCA	7	0–168 h	411	274	41	85%
12 °C	PCA	10	0–168 h	411	274	44	84%

**Table 6:** Percent of various volatile substances.

Temperature	Time/h	Alcohol/°C	Aldehyde/°C	Acid/°C	Hydrocarbon/°C	Ketone/°C	Sulfur/°C	Nitrogen/°C	Ester/°C	Phenolic/°C	Ether/°C	Other/°C	Total/°C
4 °C	0	16.617	31.289	9.968	13.962	8.135	0	2.787	5.638	0.077	0	11.527	100
	24	16.405	27.807	11.25	10.713	9.07	0	0.842	12.619	0	0.142	11.152	100
	72	14.614	26.169	4.652	18.522	7.41	0	1.534	4.588	0	1.986	20.525	100
	120	20.467	22.907	6.737	18.085	9.545	0	0.905	10.406	0	0.189	10.759	100
	168	13.597	23.676	7.045	10.841	6.594	0	1.32	16.659	0.685	0.317	19.266	100
	192	15.728	15.194	8.166	24.121	8.839	0	1.231	11.292	0.909	0.155	14.365	100
	216	15.416	16.368	11.933	16.579	4.514	12.091	1.343	11.146	1.228	0.102	9.28	100
	240	21.671	18.485	11.76	9.139	5.054	0	0	8.116	0.958	0	24.817	100
12 °C	0	16.617	31.289	9.968	13.962	8.135	0	2.787	5.638	0.077	0	11.527	100
	24	13.71	21.447	10.633	26.484	8.174	0	0.463	4.622	0	0	14.467	100
	48	12.738	22.212	12.88	14.891	6.272	0	0	13.345	0.132	0	17.53	100
	72	12.617	18.341	14.74	16.799	5.569	0	0.866	13.208	0	2.825	15.035	100
	96	19.407	16.672	15.745	11.367	8.948	0	1.004	13.302	0	1.113	12.442	100
	120	5.894	24.064	12.462	22.997	2.677	0	2.325	10.144	1.014	0	18.423	100
	144	12.855	20.484	7.309	18.405	6.94	0	3.258	12.484	1.288	0	16.977	100
	168	7.573	23.912	11.241	19.812	3.477	0	1.505	12.114	0.962	0	19.404	100
20 °C	0	16.617	31.289	9.968	13.962	8.135	0	2.787	5.638	0.077	0	11.527	100
	12	14.083	18.694	13.603	16.036	10.254	0	0	5.153	3.386	0	18.791	100
	24	13.295	20.113	17.899	14.062	10.176	0	5.7	8.208	0	0	10.547	100
	36	11.128	21.043	22.341	15.352	5.433	0	5.804	3.066	0	0	15.633	100
	48	7.417	26.368	9.418	13.441	5.697	0	0.719	4.029	0	0.294	32.617	100
	60	10.603	22.162	13.544	21.642	4.584	0	0.716	5.087	0	0	21.662	100
	72	11.494	22.22	15.432	17.869	4.052	0	1.191	4.785	0	0.133	22.824	100
28 °C	0	16.617	31.289	9.968	13.962	8.135	0	2.787	5.638	0.077	0	11.527	100
	8	15.843	14.66	8.257	20.844	8.031	0	0.969	7.404	0	0	23.992	100
	16	19.689	15.773	16.05	16.286	10.667	0	0.266	5.064	0	0	16.205	100
	24	15.164	18.193	11.966	11.737	7.789	0	1.107	5.704	0	0	28.34	100
	32	13.675	12.073	12.008	16.543	8.724	0	0	10.02	0	0	26.957	100
	40	12.939	23.773	7.828	7.074	3.845	0	0.309	18.126	0	0	26.106	100
	48	5.252	25.422	10.048	12.361	2.52	0	0.472	21.161	0	0	22.764	100

**Table 7:** Quantity of various volatile substances.

Temperature	Time/h	Alcohol	Aldehyde	Acid	Hydrocarbon	Ketone	Sulfur	Nitrogen	Ester	Phenolic	Ether	Other
4 °C	0	12	9	4	7	3	0	1	8	1	0	5
	24	9	9	6	7	4	0	2	7	0	1	5
	72	7	8	4	13	3	0	1	4	0	2	8
	120	9	13	7	12	5	0	3	13	0	1	5
	168	7	11	5	8	8	0	2	11	1	1	7
	192	9	7	8	13	7	0	2	13	2	1	7
	216	9	11	9	11	5	1	1	7	2	1	7
	240	11	12	10	7	4	0	0	6	1	0	7
12 °C	0	12	9	4	7	3	0	1	8	1	0	5
	24	11	10	5	10	4	0	1	6	0	0	8
	48	8	12	7	11	3	0	0	11	1	0	8
	72	8	9	8	13	3	0	1	10	0	1	7
	96	9	10	7	8	7	0	1	11	0	2	5
	120	5	17	8	17	1	0	2	10	1	0	5
	144	9	16	7	14	5	0	3	11	2	0	6
	168	7	15	8	12	2	0	1	10	1	0	7
20 °C	0	12	9	4	7	3	0	1	8	1	0	5
	12	6	5	5	10	2	0	0	7	1	0	6
	24	8	8	5	9	4	0	2	5	0	0	4
	36	9	9	5	11	3	0	2	5	0	0	5
	48	6	8	8	8	3	0	1	5	0	1	8
	60	8	10	8	12	4	0	1	6	0	0	4
	72	11	10	10	10	3	0	2	6	0	1	5
28 °C	0	12	9	4	7	3	0	1	8	1	0	5
	8	8	8	4	13	3	0	2	6	0	0	6
	16	11	6	9	8	7	0	1	7	0	0	9
	24	7	4	6	7	2	0	1	4	0	0	6
	32	6	4	8	8	5	0	0	6	0	0	5
	40	7	10	7	7	2	0	1	15	0	0	7
	48	5	9	7	7	3	0	1	16	0	0	5

## Data Availability

The original contributions presented in the study are included in the article/Supplementary Material, further inquiries can be directed to the corresponding authors.

## Supplementary Materials

The following supporting information can be downloaded at: https://www.mdpi.com/article/10.3390/foods13193110/s1, Tables S1–S4: Relative content of volatiles in oysters at 4 °C, 12 °C, 20 °C, 28 °C for different storage times.
