# Supplementary material for: Intelligent Evaluation and Dynamic Prediction of Oyster Freshness with Electronic Nose Based on the Distribution of Volatile Compounds Using GC–MS Analysis
Source: Foods. 2024 Sep 28;13(19):3110. doi: 10.3390/foods13193110 (PMC11475790; doi:10.3390/foods13193110)
Supplement: Supplementary file 1 [file foods-13-03110-s001.zip › foods-3192310-supplementary.pdf]

Supplementary Material

Table S1. Relative content of volatiles in oysters at 4°C for different storage times.

Number	English name	Storage time rating							
		0h	24h	72h	120h	168h	192h	216h	240h
Alcohol（**A**）									
A1	1-CYCLOOCTEN-3-OL	0.988±0.016a	--	--	--	--	0.891±0.003 b	--	0.373±0.008c
A2	2-Ethylcyclohexanol,c&t	1.084±0.011a	--	--	--	--	0.584±0.013b	--	
A3	1-Heptanol	0.866±0.008a	--	--	--	--	--	--	0.634±0.005b
A4	2,6-Cyclooctadien-1-ol	4.531±0.015c	4.974±0.006b	5.638±0.009a	2.835±0.015d	2.177±0.007e	--	1.193±0.001f	1.172±0.003g
A5	Bicyclo[3.1.1]heptan-3-ol, 2,6,6-trimethyl-, [1R-(1.alpha.,2.beta.,3.alpha.,5.alpha.)]-	1.489±0.008a	--	--	--	0.873±0.008d	--	0.955±0.017c	0.961±0.008b
A6	2-(4-Hydroxybutyl)cyclohexanol	0.810±0.006a	0.764±0.005c		0.671±0.009e	--	0.710±0.015d	--	0.794±0.009b
A7	cis,cis-7,10,-Hexadecadienal	1.487±0.008f	0.961±0.007g	1.634±0.003c	1.748±0.005b	1.558±0.003d	1.789±0.008a	1.494±0.008f	1.540±0.015e
A8	5,8,11-Heptadecatrien-1-ol	0.938±0.009a	--	--	--	--	0.920±0.006b	0.763±0.009c	--
A9	Eicosen-1-ol, cis-9-	0.973±0.011f	--	0.418±0.008g	1.411±0.001e	2.226±0.008d	3.409±0.009c	6.254±0.001b	7.144±0.003a
A10	1-Hexadecanol	0.209±0.006b	0.759±0.002a	0.112±0.001c	--	--	--	--	--
A11	Ethanol, 2-(tetradecyloxy)-	0.244±0.002b	0.880±0.005a	--	--	--	--	--	--
A12	Bicyclo[3.1.1]heptan-3-ol, 2,6,6-trimethyl-, (1.alpha.,2.beta.,3.alpha.,5.alpha.)-	--	3.059±0.002a	--	2.296±0.001c	2.411±0.009b	--	2.305±0.004c	--
A13	(-)-Myrtenol	--	1.473±0.008d	--	2.851±0.013c	3.678±0.006b	--	--	6.548±0.009a
A14	1-Octadecanol, methyl ether	--	0.165±0.013	--	--	--	--	--	--
A15	Bicyclo[4.1.0]heptane,-3-cyclopropyl,-7-hydroxymethyl, trans	--	--	4.840±0.008a	3.027±0.011b	--	2.299±0.013c	2.009±0.006d	--
A16	1-Nonanol	--	--	0.204±0.004		--	--	--	--
A17	2-Nonen-1-ol	--	--	--	0.651±0.006	--	--	--	--

A18	Isopulegol	--	--	--	4.977±0.009	--	--	--	--
A19	1-Ethynylcyclododecanol	--	--	--	--	0.674±0.016b	--	0.247±0.002c	1.631±0.009a
A20	Cyclohexanol, 5-methyl-2-(1-methylethenyl)-	--	--	--	--	--	1.800±0.006	--	--
A21	6-Pentyltetrahydro-2H-pyran-2-ol	--	--	--	--	--	3.326±0.009	--	--
A22	6-Methyl-bicyclo[4.2.0]octan-7-ol	--	--	--	--	--	--	0.196±0.006	--
A23	Tricyclo[4.2.1.1(2,5)]decan-3-ol	--	--	--	--	--	--	--	0.535±0.002
A24	9,12-Octadecadien-1-ol, (Z,Z)-	--	--	--	--	--	--	--	0.339±0.006
	**Total**	**16.617±0.011c**	**16.405±0.015d**	**14.614±0.008g**	**20.467±0.005b**	**13.597±0.006h**	**15.728±0.009e**	**15.416±0.010f**	**21.671±0.013a**
Aldehyde（B）									
B1	Nonanal	2.801±0.009a	--	0.328±0.004d	1.466±0.006b	--	--	--	0.464±0.002c
B2	2,4-Heptadienal, (E,E)-	7.875±0.002c	9.649±0.002a	8.453±0.001b	5.639±0.009d	5.385±0.002f	5.028±0.008g	4.385±0.013h	5.518±0.008e
B3	Benzaldehyde	3.815±0.005d	3.580±0.007e	3.277±0.006f	4.476±0.008b	4.807±0.006a	4.271±0.009c	1.227±0.008h	2.880±0.013g
B4	2,6-Nonadienal, (E,Z)-	10.167±0.013a	7.997±0.015c	8.906±0.001b	4.897±0.011d	4.098±0.013e	2.862±0.002f	2.815±0.006g	2.559±0.004h
B5	2-Decenal, (E)-	1.939±0.008	--	--	--	--	--	--	--
B6	Benzaldehyde, 3-ethyl-	1.139±0.002a	0.996±0.006b	1.119±0.008a	0.735±0.011c	0.731±0.008c	--	0.643±0.006d	--
B7	2,4-Decadienal, (E,E)-	1.891±0.009a	1.480±0.013d	1.615±0.016b	1.396±0.009e	1.547±0.006c	1.101±0.015h	1.159±0.011f	1.124±0.008g
B8	Tricyclo[7.1.0.0[1,3]]decane-2-carbaldehyde	0.952±0.015d	--	1.722±0.005a	0.356±0.013e	1.723±0.00814a	--	1.289±0.006c	1.353±0.009b
B9	3-(Pent-1-en-1-yl)benzaldehyde	0.710±0.006b	0.508±0.011e	0.749±0.015a	0.668±0.008c	0.710±0.006b	0.678±0.009c	0.584±0.008d	0.526±0.012e
B10	Undecanal, 2-methyl-	--	2.877±0.015a	--	--	1.908±0.006b	--	1.652±0.019c	1.014±0.006d
B11	2,4-Nonadienal, (E,E)-	--	0.348±0.006b	--	0.436±0.013a	0.423±0.013a	--	0.355±0.004b	--
B12	Heptanal	--	--	--	1.686±0.009c	1.935±0.006b	--	1.997±0.013a	--
B13	2-Octenal, (E)-	--	--	--	0.582±0.018	--	--	--	--
B14	Benzaldehyde, 2-ethyl-	--	0.372±0.009c	--	0.410±0.006a	0.409±0.002a	0.398±0.005b	--	--
B15	2-Phenoxypropanal	--	--	--	0.160±0.013	--	--	--	--

B16	Benzaldehyde, 4-ethyl-	--	--	--	--	--	0.856±0.006a	--	0.691±0.013b
B17	2,4-Decadienal, (E,Z)-	--	--	--	--	--	--	0.262±0.004b	0.281±0.009a
B18	Bicyclo[6.1.0]non-4-ene-9-carbaldehyde	--	--	--	--	--	--	--	1.826±0.006
B19	1-Cyclohexene-1-acetaldehyde, .alpha.,2-dimethyl-	--	--	--	--	--	--	--	0.249±0.005
	**Total**	**31.289±0.009a**	**27.807±0.010b**	**26.169±0.004c**	**22.907±0.012e**	**23.676±0.007d**	**15.194±0.010h**	**16.368±0.007g**	**18.485±0.003f**
Acid（C）									
C1	Dodecanoic acid	0.548±0.009a	0.361±0.010d	--	0.170±0.007f	--	0.242±0.011e	0.377±0.015c	0.404±0.008b
C2	Tetradecanoic acid	5.690±0.007a	5.048±0.013d	1.860±0.011h	3.157±0.004g	3.473±0.008f	3.684±0.002e	5.619±0.005b	5.102±0.011c
C3	n-Hexadecanoic acid	2.738±0.010e	3.935±0.011a	1.901±0.010h	2.224±0.002g	2.723±0.010f	3.007±0.008d	3.596±0.011c	3.710±0.007b
C4	Octanoic acid	--	0.160±0.013e	--	0.156±0.009f	0.175±0.015d	0.210±0.010c	0.236±0.008b	0.262±0.011a
C5	Chloroacetic acid, dodecyl ester	0.992±0.002b	1.353±0.005a	--					
C6	Acetic acid	--	0.393±0.010f	0.523±0.010c	0.435±0.005d	0.427±0.015e	0.543±0.009b	0.523±0.004c	0.640±0.015a
C7	(6Z,9Z,12Z,15Z)-Methyl octadeca-6,9,12,15-tetraenoate	--	--	0.368±0.013	--	--	--	--	--
C8	trans-Cyclohexane-1,4-dimethanol, diacetate	--	--	--	0.184±0.001	--	--	--	--
C9	Nonanoic acid	--	--	--	0.411±0.002	--	--	--	--
C10	Undec-10-ynoic acid, tridec-2-yn-1-yl ester	--	--	--	--	0.247±0.009a	0.148±0.010b	--	--
C11	Pentanoic acid	--	--	--	--	--	0.162±0.011	--	--
C12	cis-12-Octadecenoic acid methyl ester	--	--	--	--	--	0.170±0.014	--	--
C13	9,12,15-Octadecatrienoic acid, (Z,Z,Z)-	--	--	--	--	--	--	0.963±0.011a	0.900±0.013b
C14	Butanoic acid, 3-methyl-	--	--	--	--	--	--	0.200±0.013	--
C15	Tridecanoic acid	--	--	--	--	--	--	0.129±0.005b	0.145±0.009a
C16	Pentadecanoic acid	--	--	--	--	--	--	0.290±0.010a	0.276±0.002b
C17	Docosapentaenoic Acid methyl ester	--	--	--	--	--	--	--	0.194±0.008
C18	Octadecanoic acid	--	--	--	--	--	--	--	0.127±0.010

	**Total**	**9.968±0.003d**	**11.250±0.015c**	**4.652±0.008h**	**6.737±0.002g**	**7.045±0.006f**	**8.166±0.009e**	**11.933±0.010a**	**11.760±0.007b**
Hydrocarbon（D）									
D1	Styrene	2.998±0.003b	3.370±0.005a	1.768±0.001c	--	--	--	--	
D2	Tricyclo[8.6.0.0(2,9)]hexadeca-3,15-diene, trans-2,9-anti-9,10-cis-1,10	1.691±0.009c	--	--	--	--	4.166±0.010b	4.952±0.005a	--
D3	1,3-Cyclooctadiene	3..426±0.013a	--	--	--	--	1.266±0.011b	1.053±0.002c	--
D4	7-Propylidene-bicyclo[4.1.0]heptane	3.455±0.010a	--	2.987±0.008b	2.476±0.002c	--	--	--	--
D5	2-Methyl-1-nonene-3-yne	3.904±0.004a	2.723±0.011g	3.882±0.013b	3.312±0.017c	3.093±0.010e	3.310±0.007d	2.581±0.015h	2.777±0.017f
D6	Tricyclo[8.6.0.0(2,9)]hexadeca-3,15-diene, cis-2,9-anti-9,10-cis-1,10-	2.657±0.009c	2.014±0.010h	3.297±0.001a	2.694±0.008b	2.253±0.011d	2.215±0.015e	2.061±0.009g	2.124±0.002f
D7	6-[(Z)-1-Butenyl]-1,4-cycloheptadiene	1.847±0.015a	1.209±0.002c	1.229±0.010b	0.747±0.004e	--	0.944±0.005d	--	--
D8	1,5-Cyclooctadiene, 3-(1-methyl-2-propenyl)-	0.408±0.010h	0.413±0.014g	1.780±0.002c	4.431±0.013a	0.915±0.010f	1.274±0.008d	1.985±0.010b	1.270±0.007e
D9	1-Hexene, 3,3,5-trimethyl-	--	0.858±0.005	--	--	--	--	--	--
D10	Bicyclo(3.2.1)oct-2-ene	--	2.668±0.010	--	--	--	--	--	--
D11	1-Pentadecyne	--	0.828±0.002	--	--	--	--	--	--
D12	Cyclopentane, butyl-	--	--	0.404±0.012	--	--	--	--	--
D13	cis-3-Butyl-4-vinyl-cyclopentene	--	--	0.356±0.009a	--	--	3.109±0.009b	--	--
D14	1,8,11,14-Heptadecatetraene, (Z,Z,Z)-	--	--	1.113±0.004c	0.383±0.008d	2.352±0.011b	3.035±0.011a	--	--
D15	Pentalene, 1,2,3,3a,4,6a-hexahydro-	--	--	2.008±0.005a	1.405±0.010b	--	--	--	--
D16	Heptadecane	--	--	0.298±0.009f	0.346±0.015e	0.550±0.009d	0.784±0.016c	0.942±0.010b	1.496±0.011a
D17	Cyclohexene, 3-(2-methylpropyl)-	--	--	0.692±0.012a	--	--	--	--	0.635±0.013b
D18	Bicyclo[6.1.0]non-1-ene	--	--	0.260±0.002	--	--	--	--	--
D19	3-Eicosyne	--	--	0.216±0.008	--	--	--	--	--
D20	Cyclopentane, ethyl-	--	--	--	0.521±0.010a	0.458±0.009b	--	--	--

D21	Pentadecane	--	--	--	0.527±0.005	--	--	--	--
D22	Bicyclo[2.2.1]heptane, 2-(1-methylpropyl)-	--	--	--	0.239±0.009a	--	--	0.163±0.011b	--
D23	exo-Tetracyclo[5.3.1.0(2,6).0(8,10)]undecane	--	--	--	1.004±0.010a	--	--	0.676±0.009b	0.663±0.015c
D24	Cyclopentene, 5-hexyl-3,3-dimethyl-	--	--	--	--	0.108±0.013	--	--	--
D25	3-Heptadecen-5-yne, (Z)-	--	--	--	--	1.112±0.002a	--	0.900±0.014b	--
D26	1-Decene, 3,3,4-trimethyl-	--	--	--	--	--	1.776±0.010	--	--
D27	3-Octadecyne	--	--	--	--	--	0.434±0.009a	0.281±0.004b	--
D28	1H-Indene, 1-ethylidene-octahydro-, trans-	--	--	--	--	--	1.156±0.008	--	--
D29	1,4-Methanobenzocyclodecene, 1,2,3,4,4a,5,8,9,12,12a-decahydro-	--	--	--	--	--	0.652±0.005	--	--
D30	2-Methyltetracosane	--	--	--	--	--	--	1.037±0.010	--
D31	Neophytadiene	--	--	--	--	--	--	--	0.174±0.013
	**Total**	**13.962±0.006e**	**10.713±0.005g**	**18.522±0.009b**	**18.085±0.005c**	**10.841±0.006f**	**24.121±0.014a**	**16.579±0.004d**	**9.139±0.008h**
Ketone（E）									
E1	3,5-Octadien-2-one	5.140±0.014a	4.864±0.009b	4.084±0.011d	4.461±0.005c	3.200±0.002e	3.014±0.015f	0.824±0.010h	1.523±0.005g
E2	7'-Oxaspiro[cyclopropane-1,4'-tricyclo[3.3.1.0(6,8)]nonan-2'-one]	2.245±0.011c	1.633±0.002e	3.022±0.004b	3.520±0.014a	--	1.702±0.012d	--	--
E3	6-Propenylbicyclo[3.1.0]hexan-2-one	0.750±0.014e	1.158±0.001d	--	--	0.719±0.002f	1.237±0.011c	2.583±0.009b	2.946±0.004a
E4	3-Heptanone, 6-methyl-	--	1.415±0.010a	--	--	0.536±0.009b	--	0.360±0.008c	--
E5	Paroxypropione	--	--	0.304±0.009	--	--	--	--	--
E6	2-Undecanone	--	--	--	0.647±0.013c	1.325±0.015b	1.766±0.014a	--	--
E7	Spiro[adamantane-2,5'-[1.2]dioxolan]-3'-one, 4'-methylene-	--	--	--	0.399±0.005	--	--	--	--

E8	Tricyclo[3.3.0.0(2,8)]octan-3-one, 8-methyl-	--	--	--	0.518±0.010	--	--	--	--
E9	Ethanone, 1-(2-hydroxy-5-methylphenyl)-	--	--	--	--	0.184±0.002		--	--
E10	2-Tridecanone	--	--	--	--	0.155±0.002	--	--	--
E11	trans-.beta.-Ionone	--	--	--	--	0.106±0.009b	0.129±0.008a	--	--
E12	2,7-Bis(spirocyclopropane)bicyclo[2.2.1]heptan-5-one	--	--	--	--	0.369±0.013c	0.403±0.005b	0.536±0.004a	--
E13	2-Nonanone	--	--	--	--	--	0.588±0.010	--	--
E14	ortho-Hydroxypropiophenone	--	--	--	--	--	--	0.211±0.011b	0.266±0.001a
E15	Bicyclo[3.1.0]hexan-2-one, 5-(1-methylethyl)-	--	--	--	--	--	--	--	0.319±0.010
	**Total**	**8.135±0.002d**	**9.070±0.003b**	**7.410±0.008e**	**9.545±0.015a**	**6.594±0.004f**	**8.839±0.007c**	**4.514±0.011h**	**5.054±0.003g**
Sulfur-containing compound（F）									
F1	Bicyclo[3.2.1]oct-2-ene, exo-4-(phenylsulfonyl)-	--	--	--	--	--	--	12.091±0.005	--
	**Total**	--	--	--	--	--	--	**12.091±0.005**	--
Nitrogen-containing compound（G）									
G1	Oxime-, methoxy-phenyl-_	2.787±0.002	--	--	--	--	--	--	--
G2	4-Aminoheptane	--	0.431±0.009	--	--	--	--	--	--
G3	2-(E)-Hexen-1-ol, (4S)-4-amino-5-methyl-	--	0.411±0.010d	--	--	0.554±0.015c	0.687±0.009b	1.343±0.013a	--
G4	Dodecanamide	--	--	1.534±0.002a	0.642±0.005c	0.766±0.004b	0.544±0.010d	--	--
G5	Fenobucarb	--	--	--	0.226±0.010	--	--	--	--
G6	N-Cyclohexyl-2,2-diphenylacetamide	--	--	--	0.037±0.011	--	--	--	--
	**Total**	**2.787±0.002a**	**0.842±0.005e**	**1.534±0.002b**	**0.905±0.006e**	**1.320±0.006c**	**1.231±0.009d**	**1.343±0.013c**	--
Ester（H）									
H1	Formic acid, dodecyl ester	0.545±0.013b	1.267±0.011a	--	--	--	--	--	--
H2	Methyl tetradecanoate	0.499±0.010e	0.364±0.010f	0.564±0.015e	2.036±0.002a	2.048±0.004a	1.655±0.010b	1.240±0.014c	0.701±0.015d
H3	Tetradecanoic acid, ethyl ester	0.339±0.002	--	--	--	--	--	--	--
H4	Chloroacetic acid, tetradecyl ester	0.992±0.011	--	--	--	--	--	--	--

H5	Ethanol, 2-(dodecyloxy)-	1.970±0.010b	3.597±0.001a	--	--	--	--	--	--
H6	9-Octadecen-1-ol, acetate, (Z)-	0.473±0.009h	0.693±0.015g	1.770±0.002f	3.166±0.019d	3.395±0.009c	2.475±0.002e	5.124±0.019b	5.809±0.018a
H7	Fumaric acid, ethyl 2-methylallyl ester	0.529±0.025c	0.326±0.010g	0.392±0.013e	0.574±0.004b	0.923±0.002a	0.408±0.004d	0.393±0.008e	0.374±0.002f
H8	Phthalic acid, hept-4-yl isobutyl ester	0.291±0.002	--	--	--	--	--	--	--
H9	1-Octen-3-ol, trifluoroacetate	--	6.091±0.009a	--	--	4.178±0.005b	--	2.835±0.015c	--
H10	1,2-Benzenedicarboxylic acid, bis(2-methylpropyl) ester	--	0.281±0.002	--	--	--	--	--	--
H11	E-6-Octadecen-1-ol acetate	--	--	1.862±0.010b	--	2.639±0.011a	0.783±0.001c	--	--
H12	Pentanoic acid, 2-methyl-, anhydride	--	--	--	0.177±0.015	--	--	--	--
H13	Dodecanoic acid, methyl ester	--	--	--	0.124±0.010b	0.152±0.008a	--	--	--
H14	Z-(13,14-Epoxy)tetradec-11-en-1-ol acetate	--	--	--	0.683±0.008a	0.588±0.013c	0.595±0.002b	--	--
H15	Hexadecanoic acid, methyl ester	--	--	--	0.607±0.002a	0.549±0.010b	0.531±0.005c	0.446±0.011d	0.364±0.010e
H16	Methyl 4,7,10,13-hexadecatetraenoate	--	--	--	0.226±0.018b	--	--	--	0.645±0.008a
H17	7,10-Hexadecadienoic acid, methyl ester	--	--	--	0.760±0.004b	0.795±0.009a	0.646±0.010c	--	--
H18	(6Z,9Z,12Z,15Z)-Methyl octadeca-6,9,12,15-tetraenoate	--	--	--	0.663±0.009a	0.507±0.002b	0.385±0.013c	--	--
H19	9-Octadecenoic acid (Z)-, 2,3-dihydroxypropyl ester	--	--	--	0.938±0.010a	0.885±0.005b	--	--	--
H20	Linoleyl acetate	--	--	--	0.221±0.002	--	--	--	--
H21	Methyl 2-hydroxy-octadeca-9,12,15-trienoate	--	--	--	0.231±0.005	--	--	--	--
H22	Formic acid, heptyl ester	--	--	--	--	--	0.485±0.009b	0.496±0.010a	--
H23	cis-Cyclohexane-1,4-dimethanol, diacetate	--	--	--	--	--	0.187±0.002	--	--
H24	11-Tetradecen-1-ol, acetate, (Z)-	--	--	--	--	--	1.087±0.010	--	--
H25	E-10-Methyl-11-tetradecen-1-ol propionate	--	--	--	--	--	1.075±0.015	--	--

H26	Oleic acid, butyl ester	--	--	--	--	--	0.744±0.004a	0.612±0.010b	--
H27	10-Methyl-Z-11-tridecen-1-ol acetate	--	--	--	--	--	0.236±0.009	--	--
H28	.gamma.-Dodecalactone	--	--	--	--	--	--	--	0.223±0.004
	**Total**	**5.638±0.001g**	**12.619±0.009b**	**4.588±0.006h**	**10.406±0.005e**	**16.659±0.003a**	**11.292±0.006c**	**11.146±0.011d**	**8.116±0.013f**
Phenol （I）									
I1	Phenol, 4-(1,1-dimethylpropyl)-	0.077±0.010b	--	--	--	--	0.109±0.015a	--	--
I2	Phenol, 4-(1-methylpropyl)-	--	--	--	--	0.685±0.002c	0.800±0.014b	0.997±0.005a	0.958±0.010a
I3	2,5-Diethylphenol	--	--	--	--	--	--	0.231±0.010	--
	**Total**	**0.077±0.010e**	--	--	--	**0.685±0.002d**	**0.909±0.003c**	**1.228±0.007a**	**0.958±0.010b**
Ether （K）									
K1	Hexyl octyl ether	--	0.142±0.010	--	--	--	--	--	--
K2	1,2-Epoxynonane	--	--	1.676±0.002	--	--	--	--	--
K3	Octane, 1,1'-oxybis-	--	--	0.310±0.007	--	--	--	--	--
K4	2-Ethyl-4-methylanisole	--	--	--	0.189±0.010	--	--	--	--
K5	Dodecyl nonyl ether	--	--	--	--	0.317±0.013	--	--	--
K6	Benzene, (2-methoxyethenyl)-	--	--	--	--	--	0.155±0.002	--	--
K7	Decyl octyl ether	--	--	--	--	--	--	0.102±0.009	--
	**Total**	--	**0.142±0.010e**	**1.986±0.004a**	**0.189±0.010c**	**0.317±0.013b**	**0.155±0.002d**	**0.102±0.009f**	--
Else （J）									
J1	cis-2-(2-Pentenyl)furan	6.235±0.013d	--	4.913±0.015f	5.674±0.002e	8.045±0.001c	9.033±0.008b	--	16.084±0.002a
J2	Furan, 2-ethyl-	0.678±0.005c	--	0.876±0.009a	0.670±0.007c	0.715±0.010b	0.879±0.009a	0.624±0.013d	0.310±0.007e
J3	3-Oxatricyclo[3.2.1.0(2,4)]octane, (1.alpha.,2.beta.,4.beta.,5.alpha.)-	2.962±0.010b	3.706±0.011a	--	--	2.652±0.015c	--	--	--
J4	2-(2-Isopropenyl-5-methylcyclopentylmethoxy)tetrahydropyran	0.693±0.004b	--	0.872±0.010a	0.577±0.002c	0.496±0.010e	0.523±0.011d	0.559±0.005c	--
J5	Dibenzofuran	0.959±0.002	--	--	--	--	--	--	--
J6	trans-2-(2-Pentenyl)furan	--	2.896±0.004	--	--	--	--	--	--
J7	1-Hexanol, 4-methyl-, (S)-	--	0.652±0.005	--	--	--	--	--	--

J8	2H-Pyran, 2-[(5-cyclopropy-lidenepentyl)oxy]tetrahydro-	--	0.442±0.009	--	--	--	--	--	--
J9	Spiro[cyclopropane-1,6'-[3]oxatricy-clo[3.2.1.0(2,4)]octane]	--	3.456±0.010a	--	--	2.760±0.007c	2.905±0.013b	2.416±0.004d	2.515±0.002d
J10	Furan, 2-(1-pentenyl)-, (E)-	--	--	3.046±0.002	--	--	--	--	--
J11	Acetic acid, chloro-, decyl ester	--	--	1.348±0.004	--	--	--	--	--
J12	(1R,2S,4S,5R,7R)-5-isopro-pyl-1-methyl-3,8-dioxatricy-clo[5.1.0.02,4]octane	--	--	7.842±0.010a	--	--	--	--	2.768±0.024b
J13	Bicyclo[2.2.1]heptane, 2-(1,1-dimethyl-2-propenyl)-	--	--	0.295±0.005	--	--	--	--	--
J14	5-Heptadecene, 1-bromo-	--	--	1.333±0.009b	--	--	0.702±0.008d	1.091±0.001c	1.471±0.010a
J15	Furan, 2-pentyl-	--	--	--	0.444±0.010d	1.524±0.002b	0.317±0.015e	2.007±0.010a	1.371±0.013c
J16	Z,Z-10,12-Hexadecadienal	--	--	--	3.394±0.013a	3.074±0.011b	--	1.422±0.004c	--
J17	Cyclotetrasiloxane, octame-thyl-	--	--	--	--	--	0.006±0.002	--	--
J18	Pentaleno[1,2-b]oxirene, oc-tahydro-, (1a.alpha.,1b.al-pha.,4a.beta.,5a.alpha.)-	--	--	--	--	--	--	1.109±0.010	--
J19	E-11,13-Tetradecadienal	--	--	--	--	--	--	--	0.298±0.011
	**Total**	**11.527±0.001e**	**11.152±0.005f**	**20.525±0.007b**	**10.759±0.009g**	**19.266±0.004c**	**14.365±0.009d**	**9.280±0.010h**	**24.817±0.013a**

Table S2. Relative content of volatiles in oysters at 12°C for different storage times.

Number	English name	Storage time rating							
		**0h**	**24h**	**48h**	**72h**	**96h**	**120h**	**144h**	**168h**
Alcohol（**A**）									
A1	6-Nonen-1-ol, (E)-	--	1.680±0.004	--	--	--	--	--	--
A2	1-Hexanol, 4-methyl-, (S)-	--	0.882±0.002	--	--	--	--	--	--
A3	2,6-Cyclooctadien-1-ol	4.531±0.015a	3.513±0.004b	2.790±0.004c	2.445±0.004d	1.934±0.001e	1.069±0.002f	0.884±0.002g	0.862±0.002h

A 4	(-)-Isopinocampheol	--	1.280±0.004a	0.999±0.002d	0.802±0.003f	0.828±0.002e	1.055±0.004c	--	1.061±0.004b
A 5	(-)-Myrtenol	--	2.110±0.004d	2.570±0.004b	2.407±0.006c	5.031±0.004a	--	1.562±0.003e	--
A 6	(+)-isopinocampheol	--	1.653±0.003d	1.918±0.007c	1.587±0.005e	2.224±0.003a	--	1.999±0.001b	--
A 7	5,8,11-Heptadecatrien-1-ol	0.938±0.009c	0.829±0.001d	--	--	--	--	1.165±0.003a	1.075±0.003b
A 8	5-Nonadecen-1-ol	--	1.218±0.002	--	--	--	--	--	--
A 9	Eicosen-1-ol, cis-9-	0.973±0.011fg	0.178±0.005h	2.568±0.004d	2.836±0.004a	1.814±0.004f	2.636±0.002c	2.677±0.007b	2.519±0.002e
A 10	1-Hexadecanol	0.209±0.006c	0.105±0.004d	0.681±0.003b	0.926±0.002a	--	--	--	0.101±0.003d
A 11	Ethanol, 2-(tetradecyloxy)-	0.244±0.002b	0.262±0.004a	--	--	--	--	--	--
A 12	2-Nonen-1-ol	--	--	0.888±0.004a	0.831±0.002b	0.688±0.004c	--	0.425±0.003d	--
A 13	1-Nonanol	--	--	0.324±0.002	--	--	--	--	--
A 14	Phytol	--	--	--	0.783±0.004	--	--	--	--
A 15	6-Pentyltetrahydro-2H-pyran-2-ol	--	--	--	--	4.588±0.009a	--	2.931±0.004b	--
A 16	9,12-Octadecadien-1-ol, (Z,Z)-	--	--	--	--	0.260±0.001b	0.268±0.003a	--	--
A 17	1-Ethynylcyclodo-decanol	--	--	--	--	2.040±0.002a	--	1.058±0.001b	--
A 18	2-(4-Hydroxybutyl)cy-clohexanol	0.810±0.006c	--	--	--	--	0.866±0.003b	--	0.991±0.003a
A 19	13-Docosen-1-ol, (Z)-	--	--	--	--	--	--	0.154±0.001	--
A 20	1-CYCLOOCTEN-3-OL	0.988±0.016a	--	--	--	--	--	--	0.964±0.002b
A 21	2-Ethylcyclohexanol,c&t	1.084±0.011	--	--	--	--	--	--	--
A 22	1-Heptanol	0.866±0.008	--	--	--	--	--	--	--
A 23	Bicyclo[3.1.1]heptan-3-ol, 2,6,6-trimethyl-, [1R-(1.alpha.,2.beta.,3.al-pha.,5.alpha.)]-	1.489±0.008	--	--	--	--	--	--	--
	**Total**	**16.617±0.011b**	**13.71±0.003c**	**12.738±0.004e**	**12.617±0.004f**	**19.407±0.006a**	**5.894±0.004h**	**12.855±0.004d**	**7.573±0.003g**

Aldehyde（**B**）									
B1	2-Hexenal	--	0.809±0.003b	--	--	--	--	0.933±0.003a	--
B2	2-Octenal, (E)-	--	0.842±0.007b	0.650±0.002d	--	--	0.900±0.004a	0.782±0.001c	--
B3	2,4-Heptadienal, (E,E)-	7.875±0.002a	6.086±0.009b	5.453±0.004c	4.062±0.003g	4.645±0.006d	4.431±0.007e	4.031±0.001h	4.076±0.001f
B4	Benzaldehyde	3.815±0.005a	2.177±0.005e	2.480±0.007d	2.800±0.005c	3.093±0.001b	1.651±0.002g	1.618±0.002g	1.747±0.002f
B5	2,6-Nonadienal, (E,Z)-	10.167±0.013a	6.786±0.006b	4.729±0.009d	5.023±0.003c	3.045±0.002e	2.674±0.004f	--	1.973±0.004g
B6	Benzaldehyde, 2-ethyl-	--	0.494±0.002b	0.514±0.004a	0.411±0.001d	0.395±0.004e	--	0.399±0.003e	0.481±0.003c
B7	2,4-Decadienal, (E,E)-	1.891±0.009a	1.488±0.004c	1.394±0.003d	1.174±0.002g	1.319±0.003e	1.101±0.001h	1.218±0.001f	1.519±0.001b
B8	cis,cis-7,10,-Hexadecadienal	1.487±0.008f	1.687±0.007e	1.919±0.001b	--	1.806±0.001d	1.843±0.003c	2.051±0.003a	--
B9	3-(Pent-1-en-1-yl)benzaldehyde	0.710±0.006c	0.635±0.002e	0.733±0.002c	0.808±0.004a	0.682±0.002d	0.626±0.001f	0.626±0.001f	0.783±0.001b
B10	(E,Z,Z)-2,4,7-Tridecatrienal	--	0.443±0.004	--	--	--	--	--	--
B11	Undecanal, 2-methyl-	--	--	1.874±0.007a	1.443±0.003c	--	--	--	1.864±0.004b
B12	2,4-Nonadienal, (E,E)-	--	--	0.436±0.004c	--	0.352±0.001d	0.457±0.003a	0.450±0.003b	--
B13	Benzaldehyde, 3-ethyl-	1.139±0.002a	--	0.943±0.004c	--	--	0.711±0.001d	--	1.039±0.001b
B14	Z,Z-10,12-Hexadecadienal	--	--	1.087±0.004e	1.771±0.004d	--	2.165±0.003b	2.267±0.003a	2.151±0.003c
B15	Benzaldehyde, 4-ethyl-	--	--	--	0.849±0.004b	0.840±0.001c	--	0.915±0.002a	--
B16	Cinnamaldehyde, (E)-	--	--	--	--	0.495±0.002	--	--	--
B17	Heptanal	--	--	--	--	--	2.459±0.008a	--	2.148±0.003b
B18	2-Hexenal, (E)-	--	--	--	--	--	1.113±0.001a	--	0.881±0.001b
B19	Nonanal	2.801±0.009a	--	--	--	--	1.551±0.003b	--	--
B20	Hexanal, 2-ethyl-	--	--	--	--	--	0.359±0.007	--	--
B21	2,4-Decadienal, (E,Z)-	--	--	--	--	--	0.348±0.002b	--	0.439±0.002a
B22	Benzaldehyde, 3-hydroxy-	--	--	--	--	--	0.143±0.002	--	--
B23	Tricyclo[7.1.0.0[1,3]]decane-2-carbaldehyde	0.952±0.015d	--	--	--	--	1.532±0.004c	1.550±0.002b	2.842±0.006a

B24	Cyclooctanecarboxaldehyde	--	--	--	--	--	--	1.005±0.004	--
B25	2,6-Nonadienal, (E,E)-	--	--	--	--	--	--	1.929±0.003	--
B26	Benzaldehyde, 2-hydroxy-	--	--	--	--	--	--	0.234±0.001	--
B27	E-11,13-Tetradecadienal	--	--	--	--	--	--	0.476±0.003	--
B28	1-Cyclohexene-1-carboxaldehyde, 4-(1-methylethenyl)-	--	--	--	--	--	--	--	0.424±0.004
B29	2-Decenal, (E)-	1.939±0.008	--	--	--	--	--	--	--
	**Total**	**31.289±0.009a**	**21.447±0.011e**	**22.212±0.009d**	**18.341±0.004g**	**16.672±0.005h**	**24.064±0.008b**	**20.484±0.006f**	**23.912±0.005c**
Acid（**C**）									
C1	Nonanoic acid	--	0.217±0.002	--	--	--	--	--	--
C2	Dodecanoic acid	0.548±0.009bc	0.565±0.004a	0.519±0.004c	0.560±0.004b	0.413±0.003e	0.423±0.001de	0.425±0.001d	0.383±0.001f
C3	Tetradecanoic acid	5.690±0.007e	6.121±0.010d	7.138±0.008c	8.012±0.004a	7.508±0.009b	5.039±0.002f	--	3.842±0.002g
C4	n-Hexadecanoic acid	2.738±0.010h	3.517±0.005g	4.080±0.009f	4.557±0.003c	6.343±0.002a	5.042±0.009b	4.512±0.011d	4.427±0.008e
C5	Acetic acid	--	0.213±0.004d	0.160±0.009f	0.201±0.001e	--	0.607±0.003c	1.088±0.003b	1.353±0.003a
C6	Tridecanoic acid	--	--	0.279±0.004b	0.285±0.002a	0.137±0.003c	0.129±0.001d	0.137±0.001c	0.133±0.001cd
C7	Pentadecanoic acid	--	--	0.240±0.002d	0.347±0.004c	0.484±0.001a	0.464±0.003b	0.469±0.003b	0.468±0.003b
C8	n-octadecanoic acid	--	--	0.464±0.004b	0.438±0.004c	0.550±0.002a	0.419±0.001d	0.343±0.001e	0.310±0.001f
C9	Myristoleic acid	--	--	--	0.340±0.004	--	--	--	--
C10	cis-5,8,11,14,17-Eicosapentaenoic acid	--	--	--	--	0.310±0.003	--	--	--
C11	Octanoic acid	--	--	--	--	--	0.339±0.003a	0.335±0.003a	0.325±0.003b
C12	Chloroacetic acid, dodecyl ester	0.992±0.002	--	--	--	--	--	--	--
	共计	**9.968±0.003g**	**10.633±0.003f**	**12.880±0.007c**	**14.740±0.009b**	**15.745±0.003a**	**12.462±0.010d**	**7.309±0.002h**	**11.241±0.006e**
Hydrocarbon（D）									
D1	Styrene	--	4.100±0.001a	--	1.147±0.007b	--	--	--	--
D2	E,Z-3-Ethylidenecyclohexene	--	0.536±0.005a	0.456±0.004b	--	0.462±0.003b	--	--	--
D3	Cyclohexene, 3,4-diethenyl-3-methyl-	--	1.700±0.002	--	--	--	--	--	--
D4	Pentalene, 1,2,3,3a,4,6a-hexahydro-	--	2.772±0.004	--	--	--	--	--	--

D 5	Cyclohexane, 2,4-diisopropyl-1,1-dimethyl-	--	6.342±0.009	--	--	--	--	--	--
D 6	7-Propylidene-bicyclo[4.1.0]heptane	3.455±0.010a	2.766±0.004b	--	--	2.225±0.004c	--	--	--
D 7	2-Methyl-1-nonene-3-yne	3.904±0.004a	3.440±0.006c	3.582±0.006b	3.247±0.002d	--	--	--	2.719±0.007e
D 8	Tricyclo[8.6.0.0(2,9)]hexadeca-3,15-diene, cis-2,9-anti-9,10-cis-1,10-	2.657±0.009c	2.736±0.004b	2.976±0.005a	2.409±0.004d	2.233±0.001e	2.164±0.007f	2.024±0.003g	2.005±0.003h
D 9	6-[(Z)-1-Butenyl]-1,4-cycloheptadiene	1.847±0.015a	1.414±0.003b	--	--	--	--	--	0.938±0.001c
D 10	1,5-Cyclooctadiene, 3-(1-methyl-2-propenyl)-	0.408±0.010f	0.678±0.004d	0.215±0.002g	2.561±0.004a	--	1.098±0.002c	0.502±0.006e	1.924±0.005b
D 11	1,3-Cyclohexadiene, 5-ethyl-	--	--	2.101±0.004a	--	1.541±0.001b	--	--	--
D 12	cis-3-Butyl-4-vinyl-cyclopentene	--	--	0.206±0.002b	--	--	--	1.064±0.001a	--
D 13	1,8,11,14-Heptadecatetraene, (Z,Z,Z)-	--	--	0.405±0.006a	0.405±0.006a	--	--	--	--
D 14	Cyclohexene, 4-methyl-1-(1-methylethenyl)-	--	--	2.543±0.004a	2.224±0.004b	--	--	--	--
D 15	Bicyclo[2.2.1]heptane, 2-(1-methylpropyl)-	--	--	0.293±0.005	--	--	--	--	--
D 16	endo-Tetracyclo[5.3.1.0(2,6).0(8,10)]undecane	--	--	1.429±0.005a	1.119±0.003b	0.895±0.004c	--	--	--
D 17	1,4-Methanobenzocyclodecene, 1,2,3,4,4a,5,8,9,12,12a-decahydro-	--	--	0.685±0.007b	0.826±0.001a	0.697±0.003b	--	--	--
D 18	1,1'-Biphenyl, 3-methyl-	--	--	--	0.136±0.002	--	--	--	--
D 19	Cyclopentane, propyl-	--	--	--	0.171±0.002	--	--	--	--
D 20	Bicyclo(3.2.1)oct-2-ene	--	--	--	1.683±0.006	--	--	--	--
D 21	Cyclohexene,3-butyl-	--	--	--	0.390±0.001	--	--	--	--
D 22	Fluorene	--	--	--	0.481±0.002	--	--	--	--

D 23	Neophytadiene	--	--	--	--	0.437±0.003a	0.144±0.003b	--	--
D 24	1,3,5,8-Undecatetraene	--	--	--	--	--	0.151±0.001	--	--
D 25	Pentadecane	--	--	--	--	--	0.733±0.002b	--	1.088±0.002a
D 26	Pentalene, 1,2,3,3a,4,6a-hexahydro-	--	--	--	--	--	1.206±0.004	--	--
D 27	Heptadecane	--	--	--	--	--	1.119±0.008c	1.389±0.007b	1.643±0.007a
D 28	Bicyclo[6.1.0]non-1-ene	--	--	--	--	--	0.109±0.001	--	--
D 29	Tricy-clo[8.6.0.0(2,9)]hexa-deca-3,15-diene, trans-2,9-anti-9,10-cis-1,10	1.691±0.009d	--	--	--	--	5.121±0.007b	4.132±0.007c	5.337±0.003a
D 30	Cyclooctene, 3-ethenyl-	--	--	--	--	--	2.268±0.003	--	--
D 31	3-Heptadecen-5-yne, (Z)-	--	--	--	--	--	1.135±0.004b	1.155±0.005a	--
D 32	5-Pentylcyclohexa-1,3-diene	--	--	--	--	--	0.191±0.001b	0.235±0.003a	--
D 33	exo-Tetracy-clo[5.3.1.0(2,6).0(8,10)] undecane	--	--	--	--	--	0.858±0.003a	0.796±0.005b	--
D 34	Benzocyclodecene, tetradecahydro-	--	--	--	--	--	2.362±0.001	--	--
D 35	1,4,8-Dodecatriene, (E,E,E)-	--	--	--	--	--	0.564±0.003	--	--
D 36	Bicyclo[4.3.0]nonane, 2-methylene-, cis-	--	--	--	--	--	1.092±0.001	--	--
D 37	Acenaphthene	--	--	--	--	--	--	1.240±0.003a	1.232±0.003b
D 38	2-Methyltetracosane	--	--	--	--	--	--	1.658±0.003	--
D 39	Bicyclo[2.2.1]heptane, 2-methyl-	--	--	--	--	--	--	0.710±0.007	--
D 40	Spiro[2.4]heptane, 1,5-dimethyl-6-methylene-	--	--	--	--	--	--	0.404±0.003	--
D 41	1-Pentadecyne	--	--	--	--	--	--	0.363±0.001	--

D 42	Hexadecane	--	--	--	--	--	--	--	1.129±0.007
D 43	1,3-Cyclooctadiene	3..426±0.013a	--	--	--	--	--	--	1.469±0.003b
D 44	1-Nonadecene	--	--	--	--	--	--	--	0.115±0.001
D 45	1,1'-Biphenyl, 2-methyl-	--	--	--	--	--	--	--	0.213±0.004
	**Total**	**13.962±0.006g**	**26.484±0.008a**	**14.891±0.003f**	**16.799±0.005e**	**11.367±0.003h**	**22.997±0.005b**	**18.405±0.007d**	**19.812±0.003c**
Ketone（**E**）									
E1	3-Heptanone, 6-methyl-	--	1.057±0.002	--	--	--	--	--	--
E2	6-(5-Methyl-furan-2-yl)-hexan-2-one	--	1.068±0.002b	--	--	1.223±0.003a	--	--	--
E3	3,5-Octadien-2-one	5.140±0.014a	4.910±0.004b	4.512±0.004c	3.618±0.005d	3.586±0.005e	--	2.173±0.002f	0.814±0.002g
E4	6-Propenylbicy-clo[3.1.0]hexan-2-one	0.750±0.014e	1.139±0.004d	1.129±0.003d	1.215±0.001d	1.572±0.004c	2.677±0.009b	2.793±0.007a	2.663±0.005b
E5	2-Undecanone	--	--	0.631±0.004c	0.736±0.002b	0.881±0.003a	--	--	--
E6	2-Octanone	--	--	--	--	1.018±0.001	--	--	--
E7	2-Tridecanone	--	--	--	--	0.229±0.002	--	--	--
E8	Paroxypropione	--	--	--	--	0.439±0.004a	--	0.295±0.002b	--
E9	2-Decanone	--	--	--	--	--	--	1.390±0.004	--
E10	Oxacycloheptadec-8-en-2-one, (8Z)-	--	--	--	--	--	--	0.289±0.003	--
	**Total**	**8.135±0.002bc**	**8.174±0.002b**	**6.272±0.003d**	**5.569±0.004e**	**8.948±0.003a**	**2.677±0.009g**	**6.940±0.002c**	**3.477±0.003f**
Sulfur-containing compound（**F**）									
F1	--	--	--	--	--	--	--	--	--
	**Total**	**--**	**--**	**--**	**--**	**--**	**--**	**--**	**--**
Nitrogen-containing compound（**G**）									
G 1	2-(E)-Hexen-1-ol, (4S)-4-amino-5-methyl-	--	0.463±0.002e	--	0.866±0.003d	1.004±0.003c	1.507±0.004b	1.528±0.004a	1.505±0.004b
G 2	2-Oxo-1-methyl-3-iso-propylpyrazine	--	--	--	--	--	0.818±0.001	--	--
G 3	9,10-Diazatricy-clo[4.4.0.0(2,8)]dec-9-ene	--	--	--	--	--	--	1.269±0.005	--
G 4	2,3-Diazabicy-clo[2.2.1]hept-2-ene, 4-methyl-1-(pent-4-en-1-yl)-	--	--	--	--	--	--	0.461±0.004	--
G 5	Oxime-, methoxy-phe-nyl-_	2.787±0.002	--	--	--	--	--	--	--

	**Total**	**2.787±0.002b**	**0.463±0.002g**	**--**	**0.866±0.003f**	**1.004±0.003e**	**2.325±0.005c**	**3.258±0.005a**	**1.505±0.004d**
Ester（**H**）									
H 1	Methyl tetradecanoate	0.499±0.010bc	0.534±0.002a	0.508±0.004b	0.351±0.004c	0.256±0.004d	--	--	--
H 2	Ethanol, 2-(dodecyloxy)-	1.970±0.010a	1.733±0.005b	1.271±0.002c	1.227±0.004d	--	--	--	--
H 3	Methyl 4,7,10,13-hexadecatetraenoate	--	0.216±0.002d	--	--	--	0.667±0.002c	0.704±0.002b	0.763±0.002a
H 4	Z-(13,14-Epoxy)tetradec-11-en-1-ol acetate	--	0.790±0.004d	1.342±0.004a	1.244±0.002c	1.298±0.002b	--	--	--
H 5	9-Octadecen-1-ol, acetate, (Z)-	0.473±0.009h	1.016±0.004g	1.784±0.004f	2.710±0.001d	2.447±0.004e	4.718±0.007a	2.901±0.005c	4.480±0.003b
H 6	Fumaric acid, ethyl 2-methylallyl ester	0.529±0.025b	0.333±0.003f	0.421±0.001c	0.414±0.004d	1.119±0.003a	0.507±0.001bc	0.344±0.003e	0.505±0.001b
H 7	Formic acid, heptyl ester	--	--	0.543±0.002c	--	0.449±0.001e	0.589±0.003b	0.513±0.003d	0.604±0.002a
H 8	1-Octen-3-ol, trifluoroacetate	--	--	5.266±0.004a	4.754±0.004b	--	--	--	--
H 9	Tetradecanoic acid, ethyl ester	--	--	0.711±0.004b	0.824±0.003a	0.512±0.005c	0.229±0.002d	0.188±0.002e	0.154±0.002f
H 10	2- Chloropropionic acid, hexadecyl ester	--	--	0.350±0.003	--	--	--	--	--
H 11	Oleic acid, butyl ester	--	--	0.359±0.003f	0.468±0.002e	1.450±0.001b	1.188±0.005a	0.986±0.003d	0.994±0.003c
H 12	1,2-Benzenedicarboxylic acid, bis(2-methylpropyl) ester	--	--	0.375±0.002f	0.712±0.007e	0.732±0.004d	0.773±0.001c	1.037±0.004b	1.178±0.005a
H 13	11,13-Dimethyl-12-tetradecen-1-ol acetate	--	--	0.415±0.002b	0.504±0.002a	--	--	--	--
H 14	Formic acid, octyl ester	--	--	--	--	0.142±0.007b	--	--	0.212±0.001a
H 15	Chloroacetic acid, dodec-9-ynyl ester	--	--	--	--	2.713±0.003	--	--	--
H 16	11-Tetradecen-1-ol, acetate, (Z)-	--	--	--	--	2.184±0.003	--	--	--
H 17	E-6-Octadecen-1-ol acetate	--	--	--	--	--	0.659±0.002c	4.604±0.008a	2.561±0.007b
H 18	Vinyl trans-cinnamate	--	--	--	--	--	0.116±0.004	--	--
H 19	.gamma.-Dodecalactone	--	--	--	--	--	0.698±0.003a	0.616±0.002c	0.663±0.003b

H 20	11-Tetradecyn-1-ol acetate	--	--	--	--	--	--	0.388±0.003	--
H 21	6-Octen-1-ol, 3,7-dimethyl-, propanoate	--	--	--	--	--	--	0.203±0.004	--
H 22	Formic acid, dodecyl ester	0.545±0.013	--	--	--	--	--	--	--
H 23	Tetradecanoic acid, ethyl ester	0.339±0.002	--	--	--	--	--	--	--
H 24	Chloroacetic acid, tetradecyl ester	0.992±0.011	--	--	--	--	--	--	--
H 25	Phthalic acid, hept-4-yl isobutyl ester	0.291±0.002	--	--	--	--	--	--	--
	**Total**	**5.638±0.001g**	**4.622±0.006h**	**13.345±0.005a**	**13.208±0.006c**	**13.302±0.004b**	**10.144±0.006f**	**12.484±0.003d**	**12.114±0.005e**
Phenol（I）									
I1	Phenol, 4-(1,1-dimethylpropyl)-	0.077±0.010b	--	0.132±0.004a	--	--	--	--	--
I2	Phenol, 4-(1-methylpropyl)-	--	--	--	--	--	1.014±0.003b	1.060±0.007a	0.962±0.003c
I3	1,3-Benzenediol, 4-propyl-	--	--	--	--	--	--	0.228±0.001	--
	**Total**	**0.077±0.010e**	--	**0.132±0.004d**	--	--	**1.014±0.003b**	**1.288±0.005a**	**0.962±0.003c**
Ether（K）									
K 1	Oleyl alcohol, methyl ether	--	--	--	2.825±0.002a	0.172±0.003b	--	--	--
K 2	Octyl tetracosyl ether	--	--	--	--	0.941±0.001	--	--	--
	**Total**	--	--	--	**2.825±0.002a**	**1.113±0.004b**	--	--	--
Else（J）									
J1	Furan, 2-pentyl-	--	1.198±0.002g	1.427±0.004d	1.400±0.004e	1.225±0.004f	2.022±0.003c	2.274±0.003a	2.048±0.003b
J2	2-(2-Isopropenyl-5-methylcyclopentylmethoxy)tetrahydropyran	0.693±0.004b	0.675±0.004b	0.708±0.004a	0.620±0.004c	0.538±0.003d	--	--	--
J3	Furan, 2-ethyl-	--	0.616±0.002c	0.393±0.004f	0.375±0.002g	0.531±0.001e	0.631±0.002b	0.731±0.003a	0.587±0.002d
J4	2-n-Butyl furan	--	0.760±0.007a	--	--	--	--	0.357±0.004b	--
J5	3-Oxatricyclo[3.2.1.0(2,4)]octane, (1.alpha.,2.beta.,4.beta.,5.alpha.)-	2.962±0.010b	3.414±0.005a	--	--	--	--	--	--
J6	Furan, 2-(1-pentenyl)-, (E)-	--	5.541±0.005b	--	--	--	--	--	10.156±0.001a

J7	Cyanogen bromide	--	0.002±0.001	--	--	--	--	--	--
J8	7'-Oxaspiro[cyclopropane-1,4'-tricyclo[3.3.1.0(6,8)]nonan-2'-one]	2.245±0.011e	2.261±0.006d	4.101±0.007a	3.386±0.004b	3.185±0.005c	--	--	--
J9	Bicyclo[4.1.0]heptane,-3-cyclopropyl,-7-hydroxymethyl, trans	--	--	3.254±0.006a	2.893±0.007b	--	--	--	--
J10	cis-2-(2-Pentenyl)furan	--	--	6.055±0.009d	5.438±0.009e	6.963±0.010c	10.920±0.007a	9.117±0.003b	--
J11	Dibenzofuran	--	--	0.432±0.003b	--	--	--	--	0.905±0.001a
J12	Bicyclo[3.1.1]hept-3-ene-spiro-2,4'-(1',3'-dioxane), 7,7-dimethyl-	--	--	1.160±0.006	--	--	--	--	--
J13	7-Oxabicyclo[4.1.0]heptane, 3-oxiranyl-	--	--	--	0.923±0.006	--	--	--	--
J14	Spiro[cyclopropane-1,6'-[3]oxatricyclo[3.2.1.0(2,4)]octane]	--	--	--	--	--	2.556±0.005b	2.341±0.002c	2.566±0.006a
J15	Pentaleno[1,2-b]oxirene, octahydro-, (1a.alpha.,1b.alpha.,4a.beta.,5a.alpha.)-	--	--	--	--	--	2.294±0.003	--	--
J16	8-Methylene-3-oxatricyclo[5.2.0.0(2,4)]nonane	--	--	--	--	--	--	2.157±0.006a	2.142±0.004b
J17	1-Propanone, 1-(2-furanyl)-	--	--	--	--	--	--	--	1.000±0.003
J18	Furan, 2-ethyl-	0.678±0.005	--	--	--	--	--	--	--
J19	Dibenzofuran	0.959±0.002	--	--	--	--	--	--	--
	**Total**	**11.527±0.001h**	**14.467±0.007f**	**17.530±0.009c**	**15.035±0.007e**	**12.442±0.004g**	**18.423±0.004b**	**16.977±0.006d**	**19.404±0.004a**

Table S3. Relative content of volatiles in oysters at 20°C for different storage times.

Number	English name	Storage time rating						
		0h	12h	24h	36h	48h	60h	72h
Alcohol（**A**）								

A1	trans,cis-2,6-Nonadien-1-ol	--	--	--	--	--	--	0.532±0.003
A2	Ethanol, 2-(tetradecyloxy)-	0.244±0.002b	--	--	--	--	0.642±0.004a	--
A3	Ethanol, 2-(dodecyloxy)-	1.970±0.010a	1.161±0.001e	1.535±0.004c	1.184±0.007d	0.449±0.012g	1.792±0.003b	0.827±0.009f
A4	Eicosen-1-ol, cis-9-	0.973±0.011e	4.522±0.005a	4.473±0.003b	2.010±0.004c	1.407±0.003d	0.964±0.011e	0.746±0.006f
A5	Cyclohexanol, 5-methyl-2-(1-methylethenyl)-	--	--	--	--	--	1.017±0.014	--
A6	Bicyclo[3.1.1]heptan-3-ol, 2,6,6-trimethyl-, [1S-(1.alpha.,2.beta.,3.alpha.,5.alpha.)]-	--	--	1.508±0.006	--	--	--	--
A7	Bicyclo[3.1.1]heptan-3-ol, 2,6,6-trimethyl-, [1R-(1.alpha.,2.beta.,3.alpha.,5.alpha.)]-	1.489±0.008a	--	0.823±0.008b	--	--	--	--
A8	Bicyclo[3.1.1]heptan-3-ol, 2,6,6-trimethyl-, (1.alpha.,2.beta.,3.alpha.,5.alpha.)-	--	2.075±0.015b	--	1.358±0.001d	--	2.010±0.002c	3.304±0.011a
A9	9,12-Tetradecadien-1-ol, (Z,E)-	--	--	--	--	--	--	0.593±0.002
A10	9,12-Octadecadien-1-ol, (Z,Z)-	--	0.615±0.002b	--	0.710±0.011a	--	--	0.702±0.005a
A11	6-Nonen-1-ol, (E)-	--	--	0.342±0.005	--	--	--	--
A12	2-Ethylnon-1-en-3-ol	--	--	--	0.538±0.012	--	--	--
A13	2-Ethylcyclohexanol,c&t	1.084±0.011a	--	--	--	0.836±0.013b	--	0.553±0.014c
A14	2,6-Cyclooctadien-1-ol	4.531±0.015c	4.630±0.008a	3.170±0.001c	3.183±0.015c	3.452±0.018b	1.610±0.001d	1.516±0.003e
A15	1-Octen-3-ol	--	--	0.997±0.002	--	--	--	--
A16	1-Hexadecanol	0.209±0.006d	--	0.447±0.010c	0.512±0.012b	0.207±0.014d	0.904±0.015a	0.466±0.013c
A17	1-Heptanol	0.866±0.008a	--	--	0.824±0.006b	--	--	--
A18	1-Ethynylcyclododecanol	--	1.080±0.009	--	--	--	--	--
A19	1-CYCLOOCTEN-3-OL	0.988±0.016d	--	--	0.809±0.016e	1.066±0.001c	1.664±0.005b	1.748±0.010a
A20	(S)-(+)-1,2-Propanediol	--	--	--	--	--	--	0.507±0.001
A21	2-(4-Hydroxybutyl)cyclohexanol	0.810±0.006	--	--	--	--	--	--
A22	cis,cis-7,10,-Hexadecadienal	1.487±0.008	--	--	--	--	--	--
A23	5,8,11-Heptadecatrien-1-ol	0.938±0.009	--	--	--	--	--	--
	**Total**	**16.617±0.011a**	**14.083±0.007b**	**13.295±0.006c**	**11.128±0.012e**	**7.417±0.005g**	**10.603±0.011f**	**11.494±0.014d**
Aldehyde（**B**）								
B1	Z,Z-10,12-Hexadecadienal	--	--	2.190±0.012a	2.126±0.014b	0.801±0.007c	0.617±0.012d	--
B2	Undecanal, 2-methyl-	--	--	--	1.945±0.005c	3.026±0.015a	--	2.201±0.008b

B3	Tricyclo[7.1.0.0[1,3]]decane-2-carbaldehyde	0.952±0.015d	--	1.992±0.003b	2.295±0.016a	0.414±0.001f	0.807±0.004e	1.042±0.011c
B4	Nonanal	2.801±0.009a	1.864±0.010d	--	--	2.076±0.005c	2.297±0.002b	--
B5	Hexanal, 2-ethyl-	--	--	0.471±0.005	--	--	--	--
B6	Heptanal	--	--	--	--	--	2.184±0.007a	1.585±0.005b
B7	Cyclooctanecarboxaldehyde	--	--	--	--	--	1.187±0.001	--
B8	Benzaldehyde, 4-ethyl-	--	--	--	--	--	--	2.285±0.003
B9	Benzaldehyde	3.815±0.005a	2.281±0.003b	1.613±0.008f	1.815±0.019e	--	2.207±0.003c	1.979±0.011d
B10	3-(Pent-1-en-1-yl)benzaldehyde	0.710±0.006a	--	--	0.260±0.002e	0.532±0.011b	0.354±0.009d	0.503±0.005c
B11	2-Nonenal, (E)-	--	--	--	0.909±0.006b	--	--	1.102±0.001a
B12	2-Hexenal	--	--	1.231±0.009d	--	1.844±0.006a	1.652±0.004b	1.314±0.013c
B13	2,6-Nonadienal, (E,Z)-	10.167±0.013a	--	--	5.854±0.009c	6.739±0.014b	2.726±0.015d	2.544±0.006e
B14	2,6-Nonadienal, (E,E)-	--	6.605±0.001a	6.300±0.004b	--	--	--	--
B15	2,4-Heptadienal, (E,E)-	7.875±0.002c	6.371±0.004e	5.309±0.006f	4.522±0.012g	10.936±0.016a	8.131±0.009b	7.665±0.004d
B16	(6Z,9Z)-Pentadecadienal	--	1.573±0.009a	1.007±0.008c	1.317±0.001b	--	--	--
B17	2-Decenal, (E)-	1.939±0.008	--	--	--	--	--	--
B18	Benzaldehyde, 3-ethyl-	1.139±0.002	--	--	--	--	--	--
B19	2,4-Decadienal, (E,E)-	1.891±0.009	--	--	--	--	--	--
	**Total**	**31.289±0.009a**	**18.694±0.005g**	**20.113±0.009f**	**21.043±0.014e**	**26.368±0.004b**	**22.162±0.007d**	**22.220±0.001c**
Acid（**C**）								
C1	Undecanoic acid	--	0.129±0.005c	--	--	--	0.234±0.009b	0.299±0.005a
C2	Tetradecanoic acid	5.690±0.007d	6.043±0.011c	7.739±0.001b	9.313±0.004a	3.395±0.003g	4.449±0.014e	4.454±0.008f
C3	Propanoic acid	--	--	--	--	--	--	0.829±0.015
C4	Phthalic acid, hept-4-yl isobutyl ester	0.291±0.002b	--	--	--	--	0.384±0.001a	--
C5	Pentanoic acid, 3-methyl-	--	--	--	--	--	0.442±0.008b	0.485±0.005a
C6	Pentadecanoic acid	--	--	0.512±0.005b	0.644±0.003a	0.104±0.005d	--	0.201±0.006c
C7	Octanoic acid	--	0.276±0.001d	--	0.317±0.006c	0.340±0.001b	0.564±0.007a	0.570±0.002a
C8	Octadecanoic acid	--	--	--	--	--	--	0.402±0.0008
C9	n-Hexadecanoic acid	2.738±0.010g	6.613±0.003c	8.765±0.003b	11.486±0.013a	3.236±0.012f	3.404±0.004e	3.704±0.001d
C10	Heptanoic acid	--	--	--	--	0.284±0.002b	0.554±0.016a	--
C11	Dodecanoic acid	0.548±0.009d	0.542±0.015d	0.649±0.008a	0.581±0.016c	0.394±0.016e	--	0.602±0.003b
C12	Benzyl alcohol	--	--	--	--	0.161±0.014	--	--
C13	Acetic acid	--	--	0.234±0.006d	--	1.504±0.010c	3.513±0.015b	3.886±0.007a
	**Total**	**9.968±0.003f**	**13.603±0.003d**	**17.899±0.008b**	**22.341±0.013a**	**9.418±0.001g**	**13.544±0.005e**	**15.432±0.012c**
Hydrocarbon（D）								
D1	Styrene	2.998±0.003a	1.316±0.009b	1.273±0.005c	1.118±0.001d	--	--	--
D2	Cyclopentane, ethyl-	--	1.157±0.005a	--	--	1.012±0.011b	--	--
D3	Pentalene, 1,2,3,3a,4,6a-hexahydro-	--	2.210±0.002c	1.647±0.004d	--	2.556±0.016a	--	2.424±0.008b

D4	1,3-Cyclooctadiene	3..426±0.013a	0.404±0.012d	1.448±0.005c	2.486±0.008b	--	--	--
D5	Allylidenecyclohexane	--	1.720±0.004a	1.472±0.001b	--	--	--	--
D6	Cyclohexene, 4-methyl-1-(1-methylethenyl)-	--	2.123±0.015	--	--	--	--	--
D7	Tricyclo[8.6.0.0(2,9)]hexadeca-3,15-diene, cis-2,9-anti-9,10-cis-1,10-	2.657±0.009c	2.891±0.007a	--	2.872±0.004b	2.124±0.003d	2.052±0.010f	2.080±0.003e
D8	endo-Tetracyclo[5.3.1.0(2,6).0(8,10)]undecane	--	1.000±0.011a	--	0.751±0.016b	--	--	--
D9	1,5-Cyclooctadiene, 3-(1-methyl-2-propenyl)-	0.408±0.010d	0.994±0.002b	0.552±0.004c	1.093±0.009a	0.389±0.015e	0.160±0.007g	0.255±0.014f
D10	1,3,5,8-Undecatetraene	--	--	--	0.200±0.001	--	--	--
D11	1,3-Cyclohexadiene, 5-butyl-	--	--	1.434±0.007e	1.631±0.014c	1.990±0.004a	1.549±0.016d	1.906±0.014b
D12	1-Octadecyne	--	--	--	--	--	0.599±0.00a1	0.494±0.005b
D13	1,4-Cyclohexadiene, 1-methyl-	--	--	--	--	3.003±0.012	--	--
D14	1,8,11,14-Heptadecatetraene, (Z,Z,Z)-	--	--	3.366±0.006	--	--	--	--
D15	1-Pentene	--	--	--	0.082±0.014b	--	0.111±0.014a	--
D16	1H-Indene, 1-ethylideneoctahydro-, trans-	--	--	--	--	--	1.265±0.008b	1.373±0.009a
D17	5-Eicosyne	--	--	--	--	--	0.898±0.015	--
D18	5-Pentylcyclohexa-1,3-diene	--	--	--	--	1.020±0.003	--	--
D19	6-[(Z)-1-Butenyl]-1,4-cycloheptadiene	1.847±0.015a	--	0.867±0.009b	--	--	--	--
D20	Benzene, 1,3-diethyl-	--	--	--	--	1.347±0.013	--	--
D21	Benzocyclodecene, tetradecahydro-	--	--	--	1.478±0.002	--	--	--
D22	Bicyclo[2.2.1]heptane, 2,2-dimethyl-3-methylene-, (1R)-	--	--	--	1.751±0.003	--	--	--
D23	Cyclohexane, 2,4-diisopropyl-1,1-dimethyl-	--	--	--	--	--	5.052±0.012	--
D24	Cyclohexene, 3-ethyl-	--	--	--	--	--	--	3.698±0.001
D25	Cyclohexene,1-(2-propenyl)-	--	--	--	--	--	3.570±0.004	--
D26	Heptadecane	--	--	--	--	--	1.220±0.016	--

D27	Pentadecane	--	--	--	--	--	0.475±0.002b	0.533±0.013a
D28	Tricyclo[8.6.0.0(2,9)]hexadeca-3,15-diene, trans-2,9-anti-9,10-cis-1,10	1.691±0.009d	--	--	1.890±0.006c	--	4.691±0.003b	4.803±0.005a
D29	Cyclohexene, 3-pentyl-	--	--	--	--	--	--	0.303±0.001
D30	2-Dodecen-4-yne, (Z)-	--	2.221±0.004	--	--	--	--	--
D31	1,Z-5,E-7-Dodecatriene	--	--	2.003±0.008	--	--	--	--
D32	7-Propylidene-bicyclo[4.1.0]heptane	3.455±0.010	--	--	--	--	--	--
D33	2-Methyl-1-nonene-3-yne	3.904±0.004	--	--	--	--	--	--
	**Total**	**13.962±0.006ef**	**16.036±0.001c**	**14.062±0.006e**	**15.352±0.007d**	**13.441±0.012f**	**21.642±0.003a**	**17.869±0.016b**
Ketone（**E**）								
E1	Tricyclo[3.3.0.0(2,8)]octan-3-one, 8-methyl-	--	--	0.972±0.005a	0.687±0.008b	--	--	--
E2	6-Propenylbicyclo[3.1.0]hexan-2-one	0.750±0.014c	--	--	--	0.735±0.001d	2.194±0.004b	2.527±0.006a
E3	3-Heptanone, 6-methyl-	--	1.703±0.002a	--	0.716±0.009b	--	0.629±0.002c	--
E4	3,5-Octadien-2-one	5.140±0.014c	8.551±0.005a	7.811±0.003b	4.030±0.001e	4.212±0.003d	1.124±0.014f	0.831±0.001g
E5	2-Undecanone	--	--	0.518±0.002	--	--	--	--
E6	2,2,6-Trimethyl-12-oxabicyclo[8.2.1]trideca-3,6,10(13)-triene-5,11-dione	--	--	--	--	0.750±0.016a	0.637±0.005b	--
E7	1,3-Decadiene-7,9-dione	--	--	0.875±0.007	--	--	--	--
E8	(3aR,3bS,6S,6aR,6bS)-6-Isopropyl-3b-methyloctahydrocyclobuta[1,2:3,4]di[5]annulen-1(2H)-one	--	--	--	--	--	--	0.694±0.012
E9	7'-Oxaspiro[cyclopropane-1,4'-tricyclo[3.3.1.0(6,8)]nonan-2'-one]	2.245±0.011	--	--	--	--	--	--
	**Total**	**8.135±0.002c**	**10.254±0.012a**	**10.176±0.006b**	**5.433±0.004f**	**5.697±0.013d**	**4.584±0.003e**	**4.052±0.015g**
Sulfur-containing compound（**F**）								
	--	--	--	--	--	--	--	--
	**Total**	--	--	--	--	--	--	--
Nitrogen-containing compound（**G**）								
G1	Oxime-, methoxy-phenyl-_	2.787±0.002c	--	3.092±0.006b	5.384±0.003a	--	--	--
G2	Cyclopentanemethanol, .alpha.-cyclohexyl-2-nitro-	--	--	--	0.420±0.001b	--	0.716±0.003a	--

G3	3,7-Dimethyl-1,7-octadien-3-amine	--	--	2.608±0.008	--	--	--	--
G4	2-Oxo-1-methyl-3-iso-propylpyrazine	--	--	--	--	0.719±0.002b	--	0.750±0.001a
G5	1H-Imidazole	--	--	--	--	--	--	0.441±0.004
	**Total**	**2.787±0.002c**	--	**5.700±0.003b**	**5.804±0.006a**	**0.719±0.002e**	**0.716±0.003e**	**1.191±0.012d**
Ester（**H**）								
H1	Tetradecanoic acid, ethyl ester	0.339±0.002e	--	0.318±0.006f	0.420±0.005d	0.616±0.013b	0.565±0.008c	0.638±0.002a
H2	Oleic acid, butyl ester	--	--	--	--	0.263±0.006	--	--
H3	Fumaric acid, ethyl 2-methylallyl ester	0.529±0.025b	0.538±0.010a	0.543±0.007a	0.413±0.001c	0.179±0.014f	0.201±0.015e	0.263±0.009d
H4	Fumaric acid, di(cyclohex-3-enylmethyl) ester	--	0.509±0.002	--	--	--	--	--
H5	Formic acid, octyl ester	--	1.037±0.015a	--	--	1.017±0.012b	--	--
H6	Formic acid, heptyl ester	--	--	--	--	--	0.653±0.007b	0.674±0.001a
H7	E-6-Octadecen-1-ol acetate	--	0.526±0.004c	--	--	1.954±0.003a	0.621±0.001b	--
H8	Chloroacetic acid, dodecyl ester	0.992±0.002a	0.527±0.016b	--	--	--	--	--
H9	Chloroacetic acid, dodec-9-ynyl ester	--	--	4.426±0.010	--	--	--	--
H10	Carbonic acid, decyl undecyl ester	--	--	--	--	--	--	1.526±0.004
H11	9-Octadecen-1-ol, acetate, (Z)-	0.473±0.009e	1.347±0.001c	2.422±0.003a	0.977±0.017d	--	1.470±0.003b	--
H12	11-Tetradecen-1-ol, acetate, (Z)-	--	0.669±0.008d	0.499±0.013e	0.773±0.003c	--	1.577±0.015a	1.255±0.016b
H13	1,2-Benzenedicarboxylic acid, bis(2-methylpropyl) ester	--	--	--	0.483±0.013a	--	--	0.429±0.006b
H14	Formic acid, dodecyl ester	0.545±0.013	--	--	--	--	--	--
H15	Methyl tetradecanoate	0.499±0.010	--	--	--	--	--	--
H16	Chloroacetic acid, tetradecyl ester	0.992±0.011	--	--	--	--	--	--
	**Total**	**5.638±0.001b**	**5.153±0.012c**	**8.208±0.005a**	**3.066±0.007g**	**4.029±0.002f**	**5.087±0.006d**	**4.785±0.004e**
Phenol（Ⅰ）								
I1	(-)-Myrtenol	--	3.386±0.003	--	--	--	--	--
I2	Phenol, 4-(1,1-dimethylpropyl)-	0.077±0.010	--	--	--	--	--	--
	**Total**	**0.077±0.010b**	**3.386±0.003a**	--	--	--	--	--
Ether（K）								

K1	Oleyl alcohol, methyl ether	--	--	--	--	0.294±0.006	--	--
K2	Diethylene glycol mono-dodecyl ether	--	--	--	--	--	--	0.133±0.002
	**Total**	--	--	--	--	**0.294±0.006a**	--	**0.133±0.002b**
Else（**J**）								
J1	Spiro[cyclopropane-1,6'-[3]oxatricy-clo[3.2.1.0(2,4)]octane]	--	--	--	--	3.129±0.002b	--	3.498±0.001a
J2	Furan, 2-propyl-	--	--	--	--	0.939±0.014	--	--
J3	Furan, 2-pentyl-	--	0.375±0.007d	--	0.343±0.016e	2.214±0.004b	5.110±0.008a	1.214±0.006c
J4	Furan, 2-hexyl-	--	--	--	--	1.333±0.001	--	--
J5	Furan, 2-ethyl-	--	0.442±0.015d	0.138±0.006f	0.306±0.012e	0.716±0.008b	0.771±0.009a	0.508±0.003c
J6	Furan, 2-(1-pentenyl)-, (E)-	--	1.843±0.001	--	--	--	--	--
J7	Furan, 2,3-dihydro-	--	--	--	--	--	1.598±0.016	--
J8	cis-2-(2-Pentenyl)furan	--	3.260±0.003e	1.607±0.008f	4.045±0.005d	13.725±0.003b	14.183±0.010a	13.057±0.004c
J9	Bicyclo[4.1.0]heptane,-3-cyclopropyl,-7-hydroxyme-thyl, trans	--	4.028±0.010a	--	3.388±0.001b	--	--	--
J10	7-Oxabicyclo[2.2.1]hept-2-ene, 5-methylene-	--	--	0.552±0.009	--	--	--	--
J11	3-Oxatricy-clo[3.2.1.0(2,4)]octane, (1.alpha.,2.beta.,4.beta.,5.al-pha.)-	2.962±0.010b	--	--	--	3.364±0.015a	--	--
J12	(1R,2S,4S,5R,7R)-5-isopro-pyl-1-methyl-3,8-dioxatri-cyclo[5.1.0.02,4]octane	--	8.843±0.006a	8.250±0.004b	7.551±0.002c	7.197±0.008d	--	4.547±0.006e
J13	Furan, 2-ethyl-	0.678±0.005	--	--	--	--	--	--
J14	2-(2-Isopropenyl-5-methyl-cyclopentylmethoxy)tetra-hydropyran	0.693±0.004	--	--	--	--	--	--
J15	Dibenzofuran	0.959±0.002	--	--	--	--	--	--
	**Total**	**11.527±0.001f**	**18.791±0.006d**	**10.547±0.003g**	**15.633±0.003e**	**32.617±0.012a**	**21.662±0.001c**	**22.824±0.008b**

Table S4. Relative content of volatiles in oysters at 28°C for different storage times.

Num ber	English name	Storage time rating						
		0h	8h	16h	24h	32h	40h	48h
Alcohol（**A**）								
A1	2,6-Cyclooctadien-1-ol	4.531±0.015c	3.728±0.007d	1.889±0.004e	6.300±0.011a	5.387±0.001b	--	--

A2	Eicosen-1-ol, cis-9-	0.973±0.011f	1.551±0.006e	2.719±0.012c	3.960±0.002a	1.937±0.015d	2.955±0.003b	0.898±0.002g
A3	1-Hexanol, 4-methyl-	--	0.868±0.012	--	--	--	--	--
A4	1-Hexadecanol	0.209±0.006f	0.302±0.008d	0.223±0.001e	0.989±0.001a	0.394±0.005c	--	0.466±0.012b
A5	(-)-Myrtenol	--	4.665±0.006b	--	--	3.695±0.002c	6.037±0.001a	--
A6	Bicyclo[3.1.1]heptan-3-ol, 2,6,6-trimethyl-, (1.alpha.,2.beta.,3.alpha.,5.alpha.)-	--	2.316±0.001b	3.654±0.003a	--	--	1.551±0.011d	1.755±0.011c
A7	Cyclohexanol, 1-ethenyl-	--	1.837±0.006	--	--	--	--	--
A8	9,12-Tetradecadien-1-ol, (Z,E)-	--	0.576±0.009	--	--	--	--	--
A9	1-Octen-3-ol	--	--	--	--	--	--	--
A10	1-Hexanol, 4-methyl-, (S)-	--	--	--	1.149±0.003	--	--	--
A11	Methanol, (1,4-dihydrophenyl)-	--	--	--	--	--	--	--
A12	9,12-Octadecadien-1-ol, (Z,Z)-	--	--	0.301±0.009	--	--	--	--
A13	1-CYCLOOCTEN-3-OL	0.988±0.016c	--	1.045±0.008b	--	--	0.606±0.002d	1.563±0.002a
A14	5-Isopropenyl-2-methyl-7-oxa-bicyclo[4.1.0]heptan-2-ol	--	--	0.770±0.005	--	--	--	--
A15	2-(4-Hydroxybutyl)cyclohexanol	0.810±0.006b	--	1.625±0.009a	--	--	--	--
A16	(9-Oxabicyclo[3.3.1]non-6-en-3-yl)methanol	--	--	5.295±0.003	--	--	--	--
A17	2-Propen-1-ol, 3-(2,6,6-trimethyl-2-cyclohexen-1-yl)-	--	--	1.723±0.004	--	--	--	--
A18	7-Hexadecyn-1-ol	--	--	0.445±0.011	--	--	--	--
A19	2-Nonen-1-ol	--	--	--	--	--	--	--
A20	1-Heptanol	0.866±0.008	--	--	--	--	--	--
A21	Cyclopropanemethanol, 2-methyl-2-(4-methyl-3-pentenyl)-	--	--	--	--	--	--	--
A22	1-Pentanol	--	--	--	0.283±0.005	--	--	--
A23	2-Ethylcyclohexanol,c&t	1.084±0.011b	--	--	1.740±0.001a	--	--	--

A24	1,3-Cyclohexadiene-1-methanol, 4-(1-methylethyl)-	--	--	--	0.743±0.001	--	--	--
A25	Bicyclo[3.1.1]heptan-3-ol, 2,6,6-trimethyl-, [1R-(1.alpha.,2.beta.,3.alpha.,5.alpha.)]-	1.489±0.008	--	--	--	--	--	--
A26	Benzyl alcohol	--	--	--	--	--	--	--
A27	3-Cyclohexene-1-methanol	--	--	--	--	1.484±0.005	--	--
A28	Cyclohexanol, 2-(2-propynyloxy)-, trans-	--	--	--	--	0.778±0.009	--	--
A29	2,3-Butanediol, [R-(R*,R*)]-	--	--	--	--	--	--	--
A30	1-Nonanol	--	--	--	--	--	1.192±0.007	--
A31	(S)-(+)-1,2-Propanediol	--	--	--	--	--	0.412±0.011b	0.570±0.009a
A32	Ethanol, 2-(tetradecyloxy)-	0.244±0.002a	--	--	--	--	0.186±0.001b	--
A33	2,3-Butanediol, [S-(R*,R*)]-	--	--	--	--	--	--	--
A34	Bicyclo[3.1.1]heptan-3-ol, 2,6,6-trimethyl-	--	--	--	--	--	--	--
A35	cis,cis-7,10,-Hexadecadienal	1.487±0.008	--	--	--	--	--	--
A36	5,8,11-Heptadecatrien-1-ol	0.938±0.009	--	--	--	--	--	--
	**Total**	**16.617±0.011b**	**15.843±0.010c**	**19.689±0.006a**	**15.164±0.008d**	**13.675±0.005e**	**12.939±0.012f**	**5.252±0.009g**
Aldehyde（**B**）								
B1	Heptanal	--	1.474±0.002b	--	--	--	2.615±0.010a	--
B2	2-Hexenal, (E)-	--	1.223±0.012b	--	1.134±0.012c	--	1.376±0.015a	--
B3	Nonanal	2.801±0.009a	--	--	--	--	--	2.289±0.013b
B4	Hexanal, 2-ethyl-	--	--	--	--	--	--	--
B5	2,4-Heptadienal, (E,E)-	7.875±0.002c	--	6.734±0.009e	7.743±0.007d	3.488±0.015f	7.982±0.010b	9.008±0.014a
B6	2,6-Nonadienal, (E,Z)-	10.167±0.013a	5.377±0.015d	2.699±0.002f	8.365±0.001b	6.018±0.005c	3.703±0.014e	--
B7	(6Z,9Z)-Pentadecadienal	--	2.909±0.009a	2.170±0.002b	--	--	--	--
B8	Benzaldehyde	3.815±0.005a	2.171±0.002c	1.350±0.012d	--	2.200±0.001b	--	2.163±0.013c
B9	2-n-Butylacrolein	--	0.337±0.004	--	--	--	--	--

B10	2-Propenal, 3-phenyl-	--	0.586±0.001	--	--	--	--	--
B11	3-(Pent-1-en-1-yl)benzaldehyde	0.710±0.006a	0.583±0.008c	--	--	0.367±0.007e	0.549±0.013d	0.680±0.001b
B12	2-Hexenal	--	--	0.652±0.001b	--	--	--	1.478±0.014a
B13	Z,Z-10,12-Hexadecadienal	--	--	2.168±0.003c	--	--	3.449±0.014b	4.437±0.010a
B14	2-Undecenal	--	--	--	0.951±0.001	--	--	--
B15	Benzaldehyde, 4-ethyl-	--	--	--	--	--	1.348±0.001	--
B16	Tricyclo[7.1.0.0[1,3]]decane-2-carbaldehyde	0.952±0.015a	--	--	--	--	0.609±0.012c	0.693±0.001b
B17	Undecanal	--	--	--	--	--	1.112±0.015	--
B18	2,6,6-Trimethylcyclohexa-1,4-dienecarbaldehyde	--	--	--	--	--	1.030±0.014	--
B19	2,6-Nonadienal, (E,E)-	--	--	--	--	--	--	2.549±0.014
B20	Benzaldehyde, 3-ethyl-	1.139±0.002b	--	--	--	--	--	2.125±0.009a
B21	2-Decenal, (E)-	1.939±0.008	--	--	--	--	--	--
B22	2,4-Decadienal, (E,E)-	1.891±0.009	--	--	--	--	--	--
	**Total**	**31.289±0.009a**	**14.660±0.004f**	**15.773±0.005e**	**18.193±0.009d**	**12.073±0.012g**	**23.773±0.009c**	**25.422±0.013b**
Acid（**C**）								
C1	Nonanoic acid	--	--	--	0.544±0.014b	0.802±0.009a		
C2	Tetradecanoic acid	5.690±0.007b	4.503±0.003e	6.568±0.006a	5.279±0.004c	4.568±0.001d	2.402±0.002f	2.058±0.007g
C3	n-Hexadecanoic acid	2.738±0.010e	2.746±0.014d	5.418±0.002a	4.779±0.014b	4.280±0.001c	1.806±0.010f	1.464±0.001g
C4	Octanoic acid	--	0.384±0.001cd	0.512±0.006a	--	0.413±0.015b	0.368±0.001d	0.395±0.014bc
C5	Dodecanoic acid	0.548±0.009d	0.624±0.015c	0.385±0.014f	0.737±0.005a	0.625±0.013c	0.439±0.015e	0.667±0.001b
C6	Propanoic acid	--	--	--	0.508±0.015b	0.327±0.006d	0.344±0.014c	0.624±0.003a
C7	Acetic acid	--	--	0.610±0.001d	--	0.750±0.013c	2.255±0.010b	4.541±0.015a

C8	Doconexent	--	--	1.836±0.001	--	--	--	--
C9	Undecanoic acid	--	--	0.235±0.015	--	--	--	--
C10	Tridecanoic acid	--	--	0.078±0.003	--	--	--	--
C11	Pentadecanoic acid	--	--	0.408±0.006	--	--	--	--
C12	Hexanoic acid	--	--	--	--	--	--	--
C13	stearic acid	--	--	--	0.119±0.001	--	--	--
C14	Pentanoic acid	--	--	--	--	--	--	--
C15	Heptanoic acid	--	--	--	--	0.243±0.002b	0.214±0.001c	0.299±0.013a
C16	Pentanoic acid, 3-methyl-	--	--	--	--	--	--	--
C17	Chloroacetic acid, dodecyl ester	0.992±0.002	--	--	--	--	--	--
	**Total**	**9.968±0.003e**	**8.257±0.009f**	**16.050±0.003a**	**11.966±0.012c**	**12.008±0.002b**	**7.828±0.009g**	**10.048±0.001d**
Hydrocarbon（D）								
D1	Styrene	2.998±0.003a	0.684±0.010b	0.367±0.013c	--	--	--	--
D2	Tricyclo[8.6.0.0(2,9)]hexadeca-3,15-diene, trans-2,9-anti-9,10-cis-1,10	1.691±0.009e	1.978±0.001d	4.704±0.004a	2.395±0.008b	--	--	1.994±0.013c
D3	2-Pentadecen-4-yne, (Z)-	--	--	--	4.300±0.015a	3.220±0.001b	--	--
D4	1,3-Cyclohexadiene, 5-butyl-	--	2.670±0.005a	--	--	1.701±0.005c	1.176±0.002d	1.775±0.008b
D5	1,3-Cyclooctadiene	3..426±0.013a	1.465±0.006c	1.260±0.001d	2.381±0.003b	--	--	--
D6	1-Octadecyne	--	1.326±0.002a	1.227±0.002b	--	--	--	--
D7	3-Heptadecen-5-yne, (Z)-	--	3.769±0.004	--	--	--	--	--
D8	Trans-tricyclo[6.2.1.0(2.6)]undecane	--	2.424±0.014	--	--	--	--	--
D9	Tricyclo[8.6.0.0(2,9)]hexadeca-3,15-diene, cis-2,9-anti-9,10-cis-1,10-	2.657±0.009c	4.188±0.013a	--	--	3.648±0.001b	--	--
D10	6-[(Z)-1-Butenyl]-1,4-cycloheptadiene	1.847±0.015a	1.389±0.002c	1.746±0.003b	0.939±0.001e	1.047±0.015d	--	--

D11	1,5-Cyclooctadiene, 3-(1-methyl-2-propenyl)-	0.408±0.010d	0.951±0.014a	--	0.829±0.012c	0.881±0.011b	--	--
D12	Pentalene, 1,2,3,3a,4,6a-hexahydro-	--	--	--	0.495±0.008c	1.934±0.006b	--	2.117±0.001a
D13	Benzocyclodecene, tetradecahydro-	--	--	--	0.398±0.014b	1.262±0.003a	0.260±0.002c	--
D14	cis-3-Butyl-4-vinyl-cyclopentene	--	--	--	--	--	--	2.378±0.009
D15	Bicyclo[2.2.1]heptane, 2-(1-methylpropyl)-	--	--	0.727±0.002	--	--	--	--
D16	2-Methyl-1-nonene-3-yne	3.904±0.004a	--	2.448±0.011b	--	--	--	--
D17	1,4-Cyclooctadiene, (Z,Z)-	--	--	3.807±0.013	--	--	--	--
D18	Cyclopentane, pentyl-	--	--	--	--	2.850±0.006	--	--
D19	1,8,11,14-Heptadecatetraene, (Z,Z,Z)-	--	--	--	--	--	1.746±0.015b	1.962±0.014a
D20	2,3-Dimethyl-1-hexene	--	--	--	--	--	1.884±0.008a	1.593±0.015b
D21	Cyclohexene, 3-ethenyl-	--	--	--	--	--	1.422±0.005	--
D22	Cyclobutane, 1,2-diethenyl-3,4-dimethyl-	--	--	--	--	--	0.350±0.009	--
D23	1,8,11-Heptadecatriene, (Z,Z)-	--	--	--	--	--	0.236±0.012	--
D24	1-Methylcyclohexa-2,4-diene	--	--	--	--	--	--	0.542±0.010
D25	7-Propylidene-bicyclo[4.1.0]heptane	3.455±0.010	--	--	--	--	--	--
	**Total**	**13.962±0.006d**	**20.844±0.013a**	**16.286±0.005c**	**11.737±0.007f**	**16.543±0.014b**	**7.074±0.008g**	**12.361±0.014e**
Ketone（**E**）								
E1	3,5-Octadien-2-one	5.140±0.014d	6.742±0.010a	5.451±0.014c	6.275±0.009b	2.243±0.015f	2.933±0.002e	0.956±0.011g
E2	2-Undecanone	--	0.577±0.001b	--	--	0.606±0.012a	--	--
E3	Tricyclo[3.3.0.0(2,8)]octan-3-one, 8-methyl-	--	0.712±0.002c	1.283±0.005a	--	1.120±0.006b	--	--
E4	2-Nonanone	--	--	0.873±0.013c	1.514±0.001a	1.365±0.007b	--	--
E5	3-Heptanone, 6-methyl-	--	--	0.580±0.001	--	--	--	--

E6	6-(5-Methyl-furan-2-yl)-hexan-2-one	--	--	1.494±0.013	--	--	--	--
E7	Bicyclo[3.1.0]hex-3-en-2-one, 5-(1-methylethyl)-	--	--	0.551±0.015	--	--	--	--
E8	1H-Pyrrole-2,5-dione, 3-ethyl-4-methyl-	--	--	0.435±0.003	--	--	--	--
E9	4-Hydroxy-2,4,5-trimethyl-2,5-cyclohexadien-1-one	--	--	--	--	3.390±0.012	--	--
E10	6-Propenylbicyclo[3.1.0]hexan-2-one	0.750±0.014c	--	--	--	--	0.912±0.015b	1.273±0.002a
E11	Ethanone, 1-(2-hydroxy-5-methylphenyl)-	--	--	--	--	--	--	0.291±0.011
	**Total**	**8.135±0.002c**	**8.031±0.010d**	**10.667±0.025a**	**7.789±0.019e**	**8.724±0.004b**	**3.845±0.009f**	**2.520±0.009g**
Sulfur-containing compound（**F**）								
F1	--	--	--	--	--	--	--	--
	**Total**	--	--	--	--	--	--	--
Nitrogen-containing compound（**G**）								
G1	3-Butyn-2-amine, 2-methyl-	--	0.668±0.002	--	--	--	--	--
G2	1H-Pyrazole, 3-methyl-	--	0.301±0.001a	0.266±0.001b	--	--	--	--
G3	7-Oxabicyclo[2.2.1]heptan-2-ylmethanamine	--	--	--	1.107±0.002	--	--	--
G4	Cyclopentanemethanol, .alpha.-cyclohexyl-2-nitro-	--	--	--	--	--	0.309±0.004b	0.472±0.008a
G5	Oxime-, methoxy-phenyl-_	2.787±0.002	--	--	--	--	--	--
	**Total**	**2.787±0.002a**	**0.969±0.001c**	**0.266±0.001f**	**1.107±0.002b**	--	**0.309±0.004e**	**0.472±0.008d**
Ester（**H**）								
H1	E-6-Octadecen-1-ol acetate	--	--	--	--	2.652±0.002a	0.83±0.015b	--
H2	Ethanol, 2-(dodecyloxy)-	1.970±0.010b	1.567±0.002d	--	2.097±0.010a	1.898±0.001c	0.801±0.002e	0.811±0.014f
H3	Fumaric acid, ethyl 2-methylal-lyl ester	0.529±0.025d	0.741±0.001c	0.956±0.014a	0.404±0.002e	0.786±0.005b	0.220±0.013f	0.216±0.015fg
H4	Tetradecanoic acid, ethyl ester	0.339±0.002f	0.422±0.014d	0.762±0.002c	--	0.349±0.003e	5.711±0.001a	4.924±0.007b
H5	11-Tetradecen-1-ol, acetate, (Z)-	--	3.019±0.003a	1.442±0.003c	--	--	--	1.907±0.014b
H6	9-Octadecen-1-ol, acetate, (Z)-	0.473±0.009e	1.322±0.009d	--	2.420±0.001c	2.862±0.001b	2.857±0.014b	4.048±0.003a

H7	Phthalic acid, hept-4-yl isobutyl ester	0.291±0.002b	0.333±0.007a	--	--	--	--	--
H8	Fumaric acid, di(cyclohex-3-enylmethyl) ester	--	--	--	0.783±0.012	--	--	--
H9	Undec-10-ynoic acid, 3-methyl-but-2-en-1-yl ester	--	--	--	--	1.473±0.009	--	--
H10	Dihydro-3-methylene-5-methyl-2-furanone	--	--	0.475±0.009a	--	--	--	0.342±0.019b
H11	Carbonic acid, eicosyl vinyl ester	--	--	0.903±0.005	--	--	--	--
H12	Methyl tetradecanoate	0.499±0.010b	--	0.393±0.002c	--	--	0.292±0.015d	0.639±0.002a
H13	Hexadecanoic acid, ethyl ester	--	--	0.133±0.007c	--	--	1.443±0.010a	1.268±0.015b
H14	Undecanoic acid, ethyl ester	--	--	--	--	--	0.896±0.002b	1.089±0.017a
H15	Ethyl 9,12-hexadecadienoate	--	--	--	--	--	1.180±0.002a	0.700±0.014b
H16	Oleic acid, butyl ester	--	--	--	--	--	2.412±0.001a	1.535±0.021b
H17	Octanoic acid, ethyl ester	--	--	--	--	--	0.517±0.015b	0.843±0.015a
H18	Ethyl tridecanoate	--	--	--	--	--	0.352±0.004	--
H19	Ethyl 13-methyl-tetradecanoate	--	--	--	--	--	0.131±0.014	--
H20	Pentadecanoic acid, ethyl ester	--	--	--	--	--	0.383±0.006b	0.543±0.014a
H21	Hexadecanoic acid, methyl ester	--	--	--	--	--	0.101±0.012b	0.218±0.003a
H22	Formic acid, heptyl ester	--	--	--	--	--	--	0.765±0.001
H23	L-Proline, ethyl ester	--	--	--	--	--	--	1.313±0.010
H24	Formic acid, dodecyl ester	0.545±0.013	--	--	--	--	--	--
H25	Chloroacetic acid, tetradecyl ester	0.992±0.011	--	--	--	--	--	--
	**Total**	**5.638±0.001f**	**7.404±0.010d**	**5.064±0.003g**	**5.704±0.013e**	**10.020±0.002c**	**18.126±0.012b**	**21.161±0.003a**
Phenol（I）								
I1	Phenol, 4-(1,1-dimethylpropyl)-	0.077±0.010	--	--	--	--	--	--

	**Total**	**0.077±0.010**	--	--	--	--	--	--
Ether（K）								
K1	--	--	--	--	--	--	--	--
	**Total**	--	--	--	--	--	--	--
Else（J）								
J1	cis-2-(2-Pentenyl)furan	--	6.195±0.013e	6.490±0.002d	5.434±0.013f	9.583±0.009c	11.830±0.004b	13.605±0.002a
J2	Furan, 2-ethyl-	--	0.633±0.010b	0.441±0.014c	--	--	0.428±0.013d	0.735±0.014a
J3	(1R,2S,4S,5R,7R)-5-isopropyl-1-methyl-3,8-dioxatricyclo[5.1.0.02,4]octane	--	8.649±0.001c	--	12.596±0.001a	10.565±0.015b	3.783±0.015d	--
J4	3-Oxatricyclo[3.2.1.0(2,4)]octane, (1.alpha.,2.beta.,4.beta.,5.alpha.)-	2.962±0.010d	4.243±0.006c	--	6.138±0.002a	5.316±0.013b	--	--
J5	Furan, 2-pentyl-	--	0.967±0.012d	1.545±0.013c	--	0.579±0.001e	1.895±0.001b	2.935±0.013a
J6	Oxirane, hexadecyl-	--	3.305±0.014	--	--	--	--	--
J7	Furan, 2-propyl-	--	--	0.509±0.004b	--	--	5.822±0.002a	--
J8	1-Propanone, 1-(2-furanyl)-	--	--	1.016±0.001	--	--	--	--
J9	7'-Oxaspiro[cyclopropane-1,4'-tricyclo[3.3.1.0(6,8)]nonan-2'-one]	2.245±0.011a	--	2.158±0.015b	--	--	--	--
J10	Spiro[cyclopropane-1,6'-[3]oxatricyclo[3.2.1.0(2,4)]octane]	--	--	2.037±0.012b	--	--	--	3.762±0.004a
J11	7-Oxabicyclo[4.1.0]heptane, 3-oxiranyl-	--	--	0.778±0.002b	3.380±0.008a	--	--	--
J12	Spiro[adamantane-2,5'-[1.2]dioxolan]-3'-one, 4'-methylene-	--	--	1.231±0.006	--	--	--	--
J13	2-Oxo-1-methyl-3-isopropylpyrazine	--	--	--	--	--	0.847±0.001	--
J14	Pyridine	--	--	--	0.275±0.004	--	--	--
J15	Furan, 2-hexyl-	--	--	--	0.517±0.001c	0.914±0.004b	--	1.727±0.001a
J16	Dicyclopentadiene diepoxide	--	--	--	--	--	1.501±0.013	--
J17	Furan, 2-ethyl-	0.678±0.005	--	--	--	--	--	--

J18	2-(2-Isopropenyl-5-methylcyclopentylmethoxy)tetrahydropyran	0.693±0.004	--	--	--	--	--	--
J19	Dibenzofuran	0.959±0.002	--	--	--	--	--	--
	**Total**	**11.527±0.001g**	**23.992±0.006d**	**16.205±0.010f**	**28.340±0.008a**	**26.957±0.003b**	**26.106±0.007c**	**22.764±0.013e**
